# JMJD3 intrinsically disordered region links the 3D-genome structure to TGFβ-dependent transcription activation

**DOI:** 10.1038/s41467-022-30614-y

**Published:** 2022-06-07

**Authors:** Marta Vicioso-Mantis, Raquel Fueyo, Claudia Navarro, Sara Cruz-Molina, Wilfred F. J. van Ijcken, Elena Rebollo, Álvaro Rada-Iglesias, Marian A. Martínez-Balbás

**Affiliations:** 1 Department of Molecular Genomics, Instituto de Biología Molecular de Barcelona (IBMB), Consejo Superior de Investigaciones Científicas (CSIC), Barcelona, 08028 Spain; 2 Center for Molecular Medicine Cologne (CMMC), University of Cologne, Robert-Koch-Strasse 21, 50931 Cologne, Germany; 3 Center for Biomics, Erasmus University Medical Center Rotterdam, Rotterdam, The Netherlands; 4 Molecular Imaging Platform, Instituto de Biología Molecular de Barcelona (IBMB), Consejo Superior de Investigaciones Científicas (CSIC), Barcelona, 08028 Spain; 5 Present Address: Department of Chemical and Systems Biology, Stanford School of Medicine, Stanford University, Stanford, CA 94305 USA; 6 Present Address: Institute of Biomedicine and Biotechnology of Cantabria (IBBTEC), CSIC/University of Cantabria, Santander, Spain

**Keywords:** Chromatin structure, Transcriptional regulatory elements

## Abstract

Enhancers are key regulatory elements that govern gene expression programs in response to developmental signals. However, how multiple enhancers arrange in the 3D-space to control the activation of a specific promoter remains unclear. To address this question, we exploited our previously characterized TGFβ-response model, the neural stem cells, focusing on a ~374 kb locus where enhancers abound. Our 4C-seq experiments reveal that the TGFβ pathway drives the assembly of an enhancer-cluster and precise gene activation. We discover that the TGFβ pathway coactivator JMJD3 is essential to maintain these structures. Using live-cell imaging techniques, we demonstrate that an intrinsically disordered region contained in JMJD3 is involved in the formation of phase-separated biomolecular condensates, which are found in the enhancer-cluster. Overall, in this work we uncover novel functions for the coactivator JMJD3, and we shed light on the relationships between the 3D-conformation of the chromatin and the TGFβ-driven response during mammalian neurogenesis.

## Abstract (web summary)

Here the authors demonstrate that TGFβ drives multi-enhancer contacts and ultimately gene activation during neuronal commitment, and that this requires the intrinsically disordered region (IDR) of the histone demethylase JMJD3 likely through its role in promoting phase-separated biomolecular condensates.

## Introduction

During mammalian neurogenesis, neural stem cell (NSC) progenitors differentiate into neurons in response to different signaling pathways^1^. Upon developmental pathway activation, transcription factors are recruited to the chromatin, and together with epigenetic regulators, they activate cis-regulatory elements that will establish cell-specific gene expression patterns^2–4^. In mammals, promoters are usually regulated by more than one enhancer, and in fact, the number of enhancers in the mouse genome is one order of magnitude larger than the number of promoters^5,6^. This complex and sometimes-redundant configuration is crucial to ensure precise spatial-temporal control of the gene expression. However, how these multiple enhancers are orchestrated to regulate their target genes is still an open debate. Clusters of enhancers, also named super-enhancers by others^7^, are regions of euchromatin that are characterized by a high density of binding motifs, where transcription factors and cofactors such as Mediator, RNA-polymerase II (RNAPII) or chromatin remodelers colocalize (for review^8^). In recent times, it has been proposed that these clusters of enhancers facilitate transcriptional activation by promoting liquid-liquid phase separation (LLPS), a process by which molecules are condensed and concentrated in membrane-less compartments^9–11^. These condensates have been proposed to be formed by dynamic and weak multivalent interactions, that are characteristic of proteins that contain intrinsically disordered regions (IDR)^12–18^. Thus, intrinsically disordered proteins or regions have been suggested to drive the formation or to be incorporated into these biomolecular condensates. Despite the importance of enhancer clusters in cell identity establishment, we are still far from totally understanding the mechanisms by which they control gene transcription. Many research articles have revealed the importance of the 1D and 3D structure of the chromatin in development (reviewed in^19–22^). Nonetheless, the field lacks a specific assessment of the impact of individual developmental pathways on chromatin re-organization and function of specific loci.

To fill this gap, we have analyzed the chromatin reorganization that underlies the transforming growth factor beta (TGFβ) pathway activation during neuronal commitment. We and others have demonstrated that in response to TGFβ, neural progenitors lose multipotency and commit to the neuronal lineage both in vivo and in vitro^23–26^. To do that, SMAD2/3, the major effectors of the pathway, cooperate with specific cofactors to regulate transcription. Particularly, SMAD3 interacts with the lysine demethylase (KDM) JMJD3^23,27,28^, a Jumonji C (JmjC) domain-containing enzyme that catalyzes the histone 3 lysine 27 trimethylation (H3K27me3) removal^29,30^ and has been linked to numerous developmental processes (reviewed in^31,32^). In cortical progenitor cells, we have previously shown that JMJD3 cooperates with the TGFβ pathway to induce neuronal differentiation^23,33^. In this context, JMJD3 and SMAD3 together bind and trigger the activation of neural cis-regulatory elements, presumably guided by the pioneer lineage-specific transcription factor ASCL1, and cooperating with the chromatin remodeler CHD8^33^. Although some of the linear molecular components involved in the TGFβ-mediated enhancer activation have been identified, the relevance of their interactions at the 3D-level is still to be uncovered.

Here, we perform 4C-seq experiments, and we illustrate that TGFβ drives enhancer-enhancer contacts that facilitate an enhancer cluster assembly, and ultimately gene activation. Upon TGFβ stimulation, we observe that the establishment of multi-enhancer interactions requires the coactivator JMJD3. Using live-cell imaging and molecular biology techniques, we demonstrate that a proline-rich IDR contained in JMJD3 is essential to induce LLPS, and we report a correlation between the JMJD3-containing molecular condensates and the enhancer driven gene activation. With our work, we reveal that JMJD3, containing a disordered domain, lies at the edge of chromatin structure and function upon TGFβ stimulation of NSCs.

## Results

### TGFβ drives enhancer cluster assembly

**Fig. 1:**
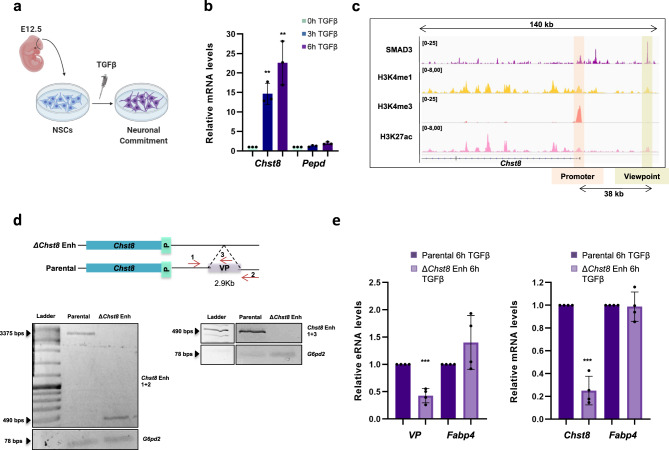
The VP is an essential *Chst8* enhancer. **a** Schematic view of the model used in this study. NSCs were dissected from cerebral cortices of mouse fetal brains (E12.5) and cultured ex vivo (see methods). TGFβ addition leads to neuronal commitment. **b** NSCs were treated with TGFβ. Total RNA was prepared and the levels of the mRNA of the indicated genes were determined by qPCR. Values were normalized to the housekeeping gene *Gapdh* and the figure shows values relative to time 0 h. Results are the mean of three biologically independent experiments. Data are presented as mean values +/− SEM. **p < 0.01 (*P* values were calculated using two-tailed Student’s *t* test, *p* = 0.001131677 and *p* = 0.006143072). Source data are provided as a Source Data file. **c** UCSC captures showing the chromatin landscape and SMAD3 binding around the *Chst8* gene promoter and the *Chst8* putative enhancer (VP) in NSCs. Tracks display ChIP-seq in NSCs treated with TGFβ (SMAD3) or untreated NSCs (H3K4me1, H3K27ac, and H3K4me3). Promoter and VP enhancer are shaded in light orange and yellow respectively. **d** Schematic representation of the CRISPR/Cas9 experimental approach used to delete the *Chst8* putative enhancer in NSCs. Two gRNAs flanking the *Chst8* enhancer region were used to create the deletion (2.9 kb). Red arrows represent primers to test the deletion. PCRs using *Chst8* deletion and *G6pd2* pairs of primers are shown at the bottom of the figure in parental and *ΔChst8* enh NSC lines. Results are representative of three independent experiments. Source data are provided as a Source Data file. **e** Parental and Δ*Chst8* enh cell lines were treated with TGFβ for 6 h. Total RNA was prepared and the levels of eRNA of the VP enhancer (left) or *Chst8* mRNA (right) were determined by qPCR. mRNA and eRNA levels of *Fapb4* were used as a control. Values were normalized to the *Gapdh* gene, and figure shows values relative to parental line. Data are presented as mean values +/− SEM. Results are representative of three biological independent experiments. ***p < 0.001 (*P* values were calculated using two-tailed Student’s *t* test, *p* = 2.2027E-05 and *p* = 1.71676E-08). Source data are provided as a Source Data file.

The three-dimensional proximity between cis-regulatory regions has been systematically described as an intrinsic feature of the genome organization^6^. Nonetheless, the impact that the genome structure exerts over its function is still an open debate^22^. Within this framework, we hypothesize that the 3D-structure of the chromatin could be playing a role in the signal-dependent regulation of the TGFβ-responsive enhancers. To test our hypothesis, we drew upon our well-characterized TGFβ model of study, the E12.5 mouse NSCs. In these cells, the TGFβ signaling pathway is moderately active under basal conditions to allow neural progenitor proliferation^23–26^; however, further TGFβ stimulation leads to the activation of hundreds of enhancers and genes that induce neuronal commitment in vitro and in vivo^23–26,33^ (Fig. 1a). Using this system, we asked whether the activation of the enhancers that occurs upon TGFβ stimulation entails 3D-chromatin changes. For this purpose, we performed 4C-seq experiments using as a viewpoint (VP) a TGFβ-regulated enhancer that lies 38 kb downstream of the carbohydrate sulfotransferase 8 (*Chst8)* gene. The rationale to select this gene was the following: first, *Chst8* is robustly upregulated upon TGFβ treatment; as indicated in Fig. 1b, the mRNA levels of the *Chst8* gene increase up to ~25-fold upon TGFβ-stimulation. Second, the distance between the VP and the *Chst8* gene allows for a reliable resolution in 4C-seq experiments (38 kb) (Fig. 1c), as it is a known-fact that one of the 4C-seq caveats is the preferential ligation of the VP with its 1D closest regions, making the contacts that appear adjacent to the bait problematic to interpret. Third, *Chst8* is a moderately long gene (138 kb), thus permitting the analysis not only of the contacts at the promoter level, but also potential interactions between the VP enhancer and the *Chst8* gene body. Before analyzing which regions contact the selected VP, we confirmed that the VP is a TGFβ-responsive *Chst8* enhancer. For that purpose, we used CRISPR/Cas9 technology to delete the VP enhancer (Fig. 1d) and measured the enhancer activity and the *Chst8* transcriptional response to TGFβ-stimulation. To this end, we evaluated the transcription of enhancer RNA (eRNA), which serves as a readout of enhancer activation^34^. Results in Fig. 1e show a remarkable decrease of both enhancer activity and *Chst8* induction upon TGFβ-stimulation in the *Chst8* enhancer-deleted (*ΔChst8* Enh) cells, demonstrating that the VP enhancer is an essential cis-regulatory element of the *Chst8* gene.

**Fig. 2:**
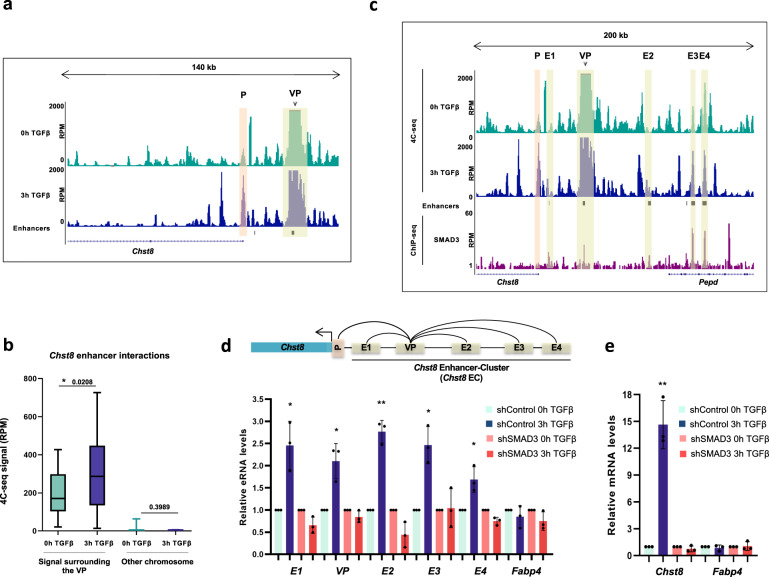
TGFβ drives enhancer-cluster assembly. **a** UCSC Genome Browser 4C-seq profiles generated in NSCs before and upon TGFβ addition are shown at the *Chst8* promoter and gene body. The light orange box indicates enhancer-promoter contact. The yellow box indicates the VP enhancer (dark arrow). **b** Boxplot displaying the averaged values obtained from two biological independent replicates of RPM signals of the peaks located 500 kb around the VP - excluding the nearest ± 20 kb - (mm10 chr7:33841896-35860773) in NSCs untreated or treated for 3 h with TGFβ. An independent region located in another chromosome (mm10 chr4:33076383-35216108) was tested as a negative control. Boxes comprise values from Q1 to Q3 of the dataset; line corresponds to median value; whiskers show the data range (from min. to max. values within dataset). Depicted quantifications were performed for n = 2 biologically independent samples. p-values are the result of a Wilcoxon-Mann–Whitney test. **c** UCSC Genome Browser captures showing 4C-seq profiles in NSCs untreated or treated (3 h) with TGFβ spanning a 200 kb distance around the VP enhancer (dark arrow). ChIP-seq signals of SMAD3 are shown. The positions of enhancers (defined in^33^) are also displayed. The light orange box indicates enhancer-promoter contacts; yellow boxes show enhancer-enhancer contacts. **d**, **e** The top panel shows a scheme summarizing the enhancer-enhancer and enhancer-promoter contacts identified in the 4C-seq experiment. The bottom panel shows the treatment of control NSCs or shSMAD3 NSCs for 3 h with TGFβ. **d** shows eRNA levels from the indicated enhancers and **e** shows mRNA from the indicated genes quantified by RT-qPCR. Transcription values were normalized to the housekeeping gene *Gapdh* and the figure shows values relative to the untreated samples. Progesterone-responsive *Fabp4* eRNA was used as a negative control. Results are the mean of three biologically independent experiments. Data are presented as mean values +/− SEM. *p < 0.05; **p < 0.01 (*P* values were calculated using two-tailed Student’s *t* test, *p* = 0.03229859 (*E1*), *p* = 0.04082807 (*VP*), *p* = 0.00839842 (*E2*), *p* = 0.02618772 (*E3*), *p* = 0.01592669 (*E4*) and *p* = 0.00113168 (*Chst8*)). Source data are provided as a Source Data file.

After testing that our selected VP is a bona fide enhancer of the *Chst8* gene, we performed two independent biological replicates of a 4C-seq experiment, where we analyzed the 3D-interactions between the VP and the genome before and after 3 h of TGFβ addition. The quality of the experiments was assessed following the criteria described in^35^ (Supplementary Data 1). The UCSC browser capture in Fig. 2a shows the obtained profiles for untreated and TGFβ treated NSCs. As expected, the proportion of cells displaying contacts between the VP enhancer and the *Chst8* promoter increased upon TGFβ treatment (see region under light orange). However, we also observed novel contacts between the VP and the *Chst8* gene body, these contacts were not particularly characterized by any type of regulatory element, but they suggest that TGFβ triggers a re-organization of the chromatin at this region (Fig. 2a and Supplementary Fig. 1a). Indeed, in the two biological replicates, the height of peaks located 500 kb upstream or downstream of the VP displayed a significant increase when NSCs cells were treated with TGFβ (p-value 0.0208), pointing to TGFβ as a driver of cis-regulatory region contacts (Fig. 2b and Supplementary Fig. 1b).

Interestingly, in addition to the contacts observed between the VP and the *Chst8* gene, we also identified several contacts occurring between the VP and inter- and intragenic enhancers located within the *Pepd* gene, a gene that lies 63 kb far from the VP, at its telomeric part (see regions under yellow in Fig. 2c). Surprisingly, the number and intensity of contacts between the VP and the enhancers located at its downstream region were higher than between the VP and the *Chst8* gene promoter. The gene *Pepd* is not regulated by TGFβ (Fig. 1b), but its intragenic VP-contacting enhancers are bound by the TGFβ transcription factor SMAD3 upon TGFβ treatment (Fig. 2c), pointing to a structural role of *Pepd* in the convergence of TGFβ-regulated enhancers that could potentially be cooperating to activate TGFβ-responsive gene promoters. These results indicate that TGFβ drives enhancer-enhancer contacts that lead to the assembly of an enhancer cluster; we named this assembly *Chst8* enhancer cluster (EC).

To confirm that the identified contacting regions within the *Chst8* EC were TGFβ-responsive enhancers, we analyzed whether they became active upon TGFβ pathway induction. To this end, we evaluated the transcription of eRNAs by qPCR upon TGFβ addition. We named the different enhancers of the EC enhancer (E)1, E2, E3, E4 and VP (Fig. 2c, d). Results in Fig. 2d show that the tested regions transcribed eRNAs in response to TGFβ. To prove the TGFβ-dependency of the EC activation we tested the eRNAs transcription in cells lacking the TGFβ pathway effector SMAD3, that were previously characterized by our lab^33^. To this end, we measured the eRNA molecules transcribed from these enhancers upon TGFβ treatment in the control cells and in the SMAD3 depleted cells (shSMAD3). In concordance with the previous results, eRNAs were hardly induced in the shSMAD3 cells compared to the control cell line (Fig. 2d). Similarly, the *Chst*8 gene was not expressed upon TGFβ addition in the shSMAD3 NSCs (Fig. 2e).

Altogether, these results demonstrate that TGFβ-driven gene activation entails a reorganization of the chromatin structure. Moreover, this reorganization results in the formation of the *Chst8* EC that is potentially involved in the regulation of genes upon TGFβ.

### TGFβ-mediated enhancer-cluster assembly depends on JMJD3

**Fig. 3:**
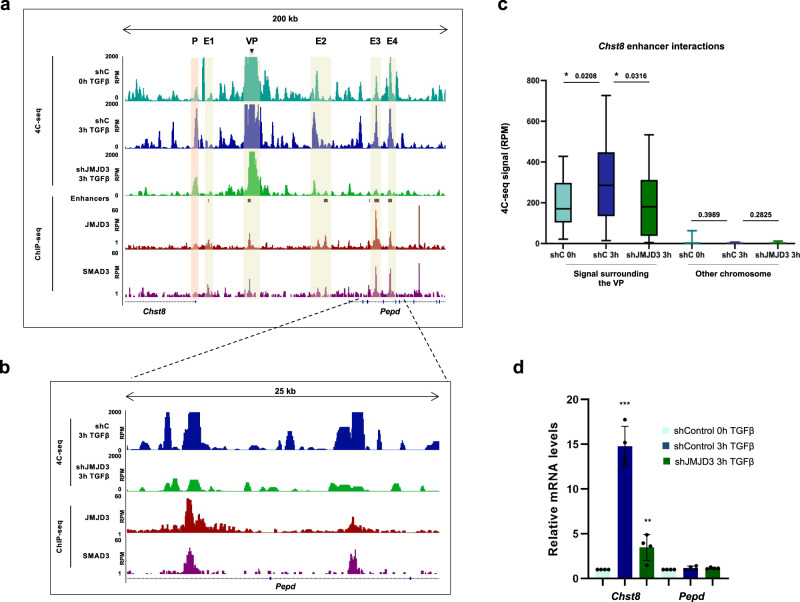
TGFβ-mediated enhancer-cluster assembly depends on JMJD3. **a** UCSC Genome Browser captures show 4C-seq profiles spanning 200 kb around the VP enhancer (black arrow) in NSCs untreated or treated (3 h) with TGFβ. ChIP-seq signals of SMAD3 and JMJD3 upon TGFβ stimulation (0.5 and 3 h, respectively) are shown. The location of the members of the EC is also indicated with yellow boxes. **b** Capture showing a zoom into a region where TGFβ-induced contacts are lost in JMJD3-depleted (shJMJD3) NSCs. **c** Boxplot displays the averaged values obtained from two biological independent replicates of RPM signals of the peaks located 500 kb around the VP - excluding the nearest ±20 kb - (mm10 chr7:33841896-35860773) in control or shJMJD3 NSCs untreated or treated with TGFβ during 3 h. An independent region located in another chromosome (mm10 chr4:33076383-35216108) was used as a negative control. *n* = 2 biologically independent replicates were quantified. p-values are the result of a Wilcoxon-Mann–Whitney test. **d** Control NSCs or shJMJD3 NSCs were treated for 3 h with TGFβ. Then, total RNA was prepared, and the levels of the mRNA of the indicated genes were determined by qPCR. Values were normalized to the housekeeping gene *Gapdh*. The figure shows values relative to time 0 h. Results are the mean of three biologically independent experiments. Data are presented as mean values +/− SEM. **p < 0.01; **p < 0.001 (*P* values were calculated using two-tailed Student’s *t* test, *p* = *p* = 0.001131677 and *p* = 0.003848794). Source data are provided as a Source Data file.

Previous work from our lab has demonstrated that the histone KDM JMJD3 functions as a cofactor for SMAD3 in the TGFβ-driven activation of neuronal enhancers in NSCs^33^. For this reason, we decided to test whether JMJD3 was also occupying the enhancers involved in the *Chst8* EC by analyzing our previously published JMJD3 ChIP-seq performed upon TGFβ stimulation^23^. As shown in Fig. 3a, all the contacting regions belonging to the *Chst8* EC (VP, E1, E2, E3 and E4) are occupied by JMJD3, consistent with the presence of enhancers at these regions. Next, motivated by the fact that the demethylase catalytic activity of JMJD3 is not involved in enhancer activation in our model^33^, we decided to address whether JMJD3 could be playing a structural role at enhancers, contributing to the *Chst8* EC assembly. For this purpose, we efficiently depleted JMJD3 from NSCs (shJMJD3 NSCs) using lentivirus containing JMJD3-specific shRNAs (Supplementary Fig. 2a and refs. ^23,33^), and then, we performed a 4C-seq assay upon TGFβ treatment using the *Chst8* VP enhancer. Figures 3a–c, and Supplementary Fig. 2b, c show the striking effect that the depletion of JMJD3 causes in the 3D-structure of the chromatin. Upon JMJD3 removal, we observed that the genomic contacts between the VP and the surrounding regions were severely reduced (Fig. 3c and Supplementary Fig. 2c). In particular, the contacts between enhancers belonging to the *Chst8* EC region were abolished upon JMJD3 depletion (Fig. 3a, b); indicating that JMJD3 is directly or indirectly required for *Chst8* EC assembly. Accordingly, *Chst8*, gene expression was markedly reduced (Fig. 3d).

To broad our conclusions and to confirm that TGFβ-driven gene activation encompasses a chromatin structure reorganization that depends on JMJD3, we analyzed the 3D-chromatin status of other two candidate enhancers that potentially regulate the TGFβ-responsive and JMJD3-dependent genes, *Ldlrad4* and *Aopep*^23^. These enhancers are located within the *Ldlrad4* and *Aopep* genes, display SMAD3 and JMJD3 binding, and are surrounded by other SMAD3/JMJD3-bound enhancers, suggesting that they could potentially engage in enhancer clusters. Indeed, 4C-seq assays using these cis-regulatory regions as VPs (see quality of the experiments assessed as described in^35^, Supplementary Data 2 and 3) show contacts between the *Ldlrad4* VP and the *Ldlrad4* promoter (Supplementary Fig. 3a, shaded in orange), and between the VPs and the surrounding enhancers (Supplementary Fig. 3a, c, shaded in yellow). In agreement with our hypothesis, upon TGFβ treatment, the frequency of the contacts between cis-regulatory elements —enhancers or promoters— increased [Supplementary Fig. 3a, b (*Ldlrad4*), c, d (*Aopep*)]. Furthermore, these contacts remarkably diminished when JMJD3 was depleted (Supplementary Fig. 3a–d). Altogether these results corroborate that the TGFβ pathway and JMJD3 are involved in 3D-chromatin structure regulation.

As JMJD3 is a coactivator^23,33,36^, we decided to rule out the possibility of an indirect transcriptional effect triggered by the lack of JMJD3 in NSCs, that could potentially be affecting the expression of the proteins involved in loop formation^37,38^. To this end, we analyzed gene expression data from our previously published microarray experiments^23^, and we show in the Supplementary Fig. 4a that neither TGFβ nor JMJD3 regulate the expression of some of the most characteristic proteins involved in loop formation (CTCF, SMC1/3, RAD21, PDS5A/B, and WAPL). Among these proteins, the Cohesin complex has been described to play a role in the establishment of dynamic contacts during gene transcription by being the main motor of the loop extrusion process^39,40^, thus, we analyzed the presence of the SMC1 subunit of the Cohesin complex in the *Chst8* EC using previously published SMC1 ChIP-seq data in NSCs^41^. Interestingly, we noticed that some of the regions occupied by JMJD3 were also bound by SMC1 (Supplementary Fig. 4b). This observation prompted us to check whether JMJD3 co-occupies genomic regions with SMC1 in a genome-wide manner. The Venn diagram in Supplementary Fig. 4c shows that these proteins do not colocalize widely across the genome (only 19% of JMJD3 peaks overlap SMC1); this can be explained by the involvement of JMJD3 in transcription elongation, that leads to many JMJD3-bound regions falling outside the cis-regulatory elements that engage in 3D-interactions^36,42^. Nonetheless, at the co-bound regions, JMJD3 and SMC1 demonstrate widespread peak overlapping (Supplementary Fig. 4d).

The results described above indicate that JMJD3 is necessary for the establishment and/or maintenance of contacts between cis-regulatory regions. JMJD3 lacks DNA binding capacity, and structurally it only has one known domain, the demethylase catalytic domain JmjC. Previous work from our laboratory has shown that its demethylase domain is not required for the activation of a subset of enhancers in response to TGFβ^33^. To deeply understand whether demethylation of the H3K27me3 is involved in the *Chst8* enhancer cluster activitation, we analyzed H3K27me3 ChIP-seq data from NSCs^36^. The results in Supplementary Fig. 5a show that the *Chst8* locus lacks H3K27me3 prior to TGFβ stimulation. Moreover, ChIP-qPCR experiments show that JMJD3 depletion did not lead to an increase in H3K27me3 levels neither at the *Chst8* promoter nor at the scrutinized enhancers (Supplementary Fig. 5b). Of note, Supplementary Fig. 5b corroborates that the levels of H3K27me3 in these regions are negligible when compared to a classic H3K27me3-controlled promoter (*Hoxd8*), suggesting that changes on the H3K27me3 levels are unlikely a force driving the *Chst8* EC formation or activation.

### JMJD3 is a highly disordered protein

**Fig. 4:**
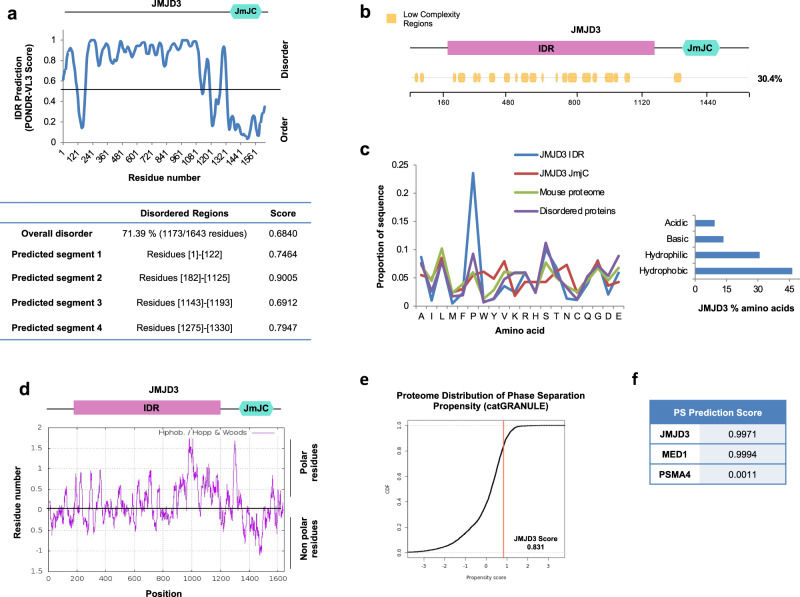
JMJD3 is a highly disordered protein. **a** Disorder prediction of human JMJD3 using PONDR-VL3 algorithm. In the bottom panel, the disorder score and the lengths of the predicted disordered regions are indicated (length of disordered segments >50 amino acids). A schematic representation of JMJD3 described domains is shown on top of the graphic. **b** Analysis of the presence of low-complexity domains in JMJD3 using the SEG algorithm. The percentage of low complexity regions is indicated on the right side. Low complexity regions are depicted in yellow. A schematic representation of JMJD3 described domains is shown on top of the panel. **c** Amino acid composition of JMJD3 IDR, JMJD3 catalytic domain, mouse proteome and disordered proteins defined by the presence of a 50 residues fragment whose IUPRED median score is at least 0.55 and that is not found in Pfam (so that functional domains are avoided). The percentages of acid, basic, hydrophobic, and hydrophilic amino acids of JMJD3 are shown on the right panel. **d** JMJD3 hydrophobicity profile was determined using the ExPASy website with the Hopp and Woods scale and a sliding window of 21. **e**, **f** The potential of JMJD3 to phase separate was determined using catGRANULE (on the left) (**e**) and PSPredictor (on the right) (**f**) algorithms.

As the investigated genomic loci are not marked by H3K27me3 prior to TGFβ activation, we hypothesized that JMJD3 could be impacting the 3D-structure of the chromatin through its unstructured domain. In the last years, numerous works have shed light on the impact on transcriptional regulation of these unstructured regions, named intrinsically disordered regions (IDR)^43,44^. With this in mind, we questioned whether JMJD3 with its unstructured domain could belong to the group of the intrinsically disordered proteins. To assess this, we analyzed the amino acid sequence of JMJD3 searching for disordered regions using the following previously validated algorithms: PONDR-VL3^45^, IUPred^46^ and VSL2^47^ (see methods). Overall, the three algorithms agreed on the highly significant disorder score of JMJD3 (Fig. 4a and Supplementary Fig. 6a, b). Specifically, PONDR-VL3 showed a median disorder score of 0.68 for JMJD3 (Fig. 4a), a value considerably higher than the 0.28 obtained when analyzing PSMA4, a well-known structured protein used as an ordered protein control (Supplementary Fig. 6b). In addition, more than 70% of JMJD3 amino acids (71.39% using PONDR-VL3) exist in disordered domains (Fig. 4a and Supplementary Fig. 6a). Again, this value is higher than the proteasome component PSMA4, used as a negative control (23.37%) (Supplementary Fig. 6b). Looking at the different disordered fragments of JMJD3, we observed a remarkably long region with no defined structure that contains 943 amino acids (residues from 182 to 1125) and that was predicted to have a disorder score of 0.90, the highest observed in our data (Fig. 4a). From now on, we will refer to this region as JMJD3 IDR.

In addition to disorder, the nature of the amino acid composition has also been shown to play an important role in IDR-mediated transcription regulation. Moreover, disordered regions frequently coincide with low-complexity domains that are biased for certain amino acids^10,13,14,18^. To check whether this was the case for JMJD3 we used the SEG algorithm^48^ (see methods) looking for JMJD3 complexity prediction. As shown in Fig. 4b, 30.4% of JMJD3 was predicted to contain low-complexity segments. Furthermore, by examining its amino acid composition, we found a remarkable abundance of prolines (24% of the total amino acids in the protein) (Fig. 4c and Supplementary Fig. 6c), that displays widespread conservation among mammals (Supplementary Fig. 6d). Strikingly, we found proline tracks as long as 20 residues (Supplementary Fig. 6c). Other structural features of the JMJD3 IDR are its high content of charged residues (21% of the protein) (Fig. 4c, d, and Supplementary Fig. 6c), its regions different to the proline tracks that also display high hydrophobicity (Fig. 4d), and its high serine content when compared to the average in the mouse proteome, and similar to other described IDRs (Fig. 4c)^15,49^. Proline residues have been described as highly hydrophobic amino acids whose concatenation generates sticky domains that bind rapidly and reversibly to other proteins^50^. It is known that hydrophobic interactions, as well as electrostatic ones, are relevant for biomolecular condensate formation. Thus, the amino acid composition of JMJD3 seemed to favor its potential to be involved in the so-called phase separation process^9,51^. Given this, we used catGRANULE^52^ and PSPredictor^53^ algorithms (see methods) to predict JMJD3 phase separation ability. Both tools returned high scores (0.83 and 0.99 respectively) for JMJD3 (Fig. 4e, f), similar to proteins known to be involved in phase separation (e.g. MED1, 0.99) and higher than the proteasome protein PSMA4 (0.001) (Fig. 4f). Altogether, these data point to JMJD3 as a highly disordered protein with the potential to undergo phase separation.

### JMJD3 undergoes LLPS in vitro and in vivo

**Fig. 5:**
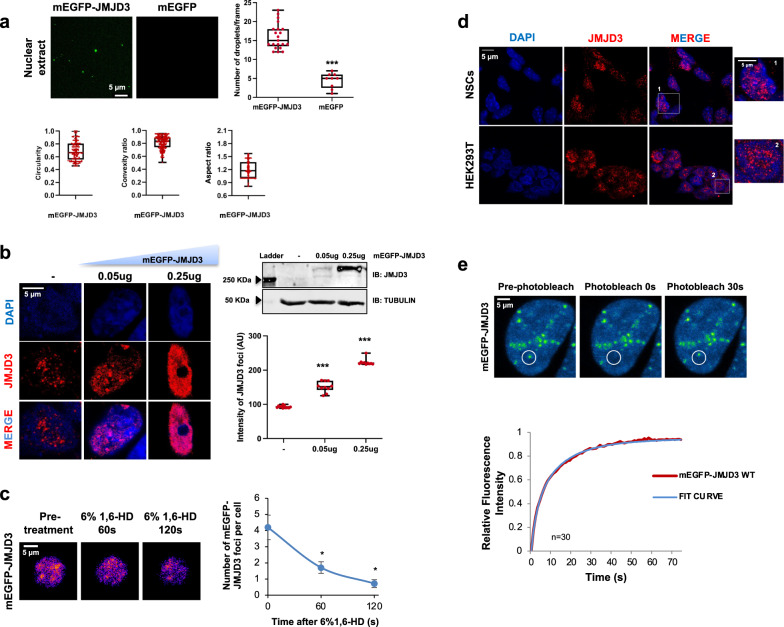
JMJD3 undergoes LLPS in vitro and in vivo. **a** mEGFP and mEGFP–JMJD3 proteins were analyzed using droplet-formation assays in nuclear extracts at room temperature with 150 mM NaCl. Quantifications of the number of droplets per frame, circularity, convexity and aspect ratio (AR) are displayed. Data are the mean ± SEM. Boxes comprise values from Q1 to Q3 of the dataset; line corresponds to median value; whiskers show the data range (from min. to max. values within dataset). ***p < 0.001 (Student’s *t* test, *p* = 1.09982E-06). Droplets in 5 fields in each group from three biologically independent experiments were quantified, *n* = 150. Scale bar, 5 μm. **b** Confocal microscopy images of HEK293T cells transfected with mEGFP-JMJD3. Quantifications of the intensity of JMJD3 puncta are shown on the right. Data show the mean ± SEM. Boxes comprise values from Q1 to Q3 of the dataset; line corresponds to median value; whiskers show the data range. ***p < 0.001 (Student’s *t* test, *p* = 9.98785E-06 and *p* = 7.15724E-17). *n* = 50 transfected cells in each group were quantified; Images are representative of 3 biologically independent experiments. Scale bar, 5 μm. Western blot displays the levels of overexpressed JMJD3. **c** HEK293T cells were transfected with 0.05 ug mEGFP–JMJD3, treated with 6% 1,6-HD for 5 min and imaged at 60 and 120 s. Nuclei were visualized with DAPI (blue). Quantification of the nuclear puncta per cell is shown on the right. Data are the mean ± SEM. *p < 0.05 (Student’s *t* test, *p* = 0.0182428 and *p* = 0.01162199). *n* = 130 transfected cells were quantified; Images are representative of three biologically independent experiments. Scale bar, 5 μm. **d** NSCs and HEK293T cells were fixed*,* and endogenous JMJD3 was visualized by immunostaining assay. The images are representative of three biologically independent experiments. Scale bar, 5 μm. **e** FRAP assay in HEK293T cells expr**e**ssing 0.05ug of mEGFP–JMJD3. Images are representative of three biological replicates. Quantification shows the curve fit results of FRAP data for mEGFP-JMJD3 to a double-exponential smoothing (R^2^ = 1), where bleaching events occurs at *t* = 0 s. Data are plot as background-subtracted and normalized mean (*n* = 27 cells). Scale bar, 5 μm. Source data are provided as a Source Data file.

LLPS is a physicochemical process that consists on the demixing of a fluid into a diluted phase and a dense phase. It is well known that proteins mediating phase separation contain IDRs, and it is starting to be uncovered the role that these IDRs play on transcription regulation as mediators of biomolecular condensation^12–15,17,54–56^. On the grounds of these recent discoveries, we hypothesized that JMJD3 could be contributing to the establishment of 3D-contacts by nucleating protein and nucleic acid scaffolds to form membrane-less condensates through LLPS. To test this idea, we decided to perform in vitro droplet assays using a construct that expressed JMJD3 fused to monomeric EGFP (mEGFP) and HA (Supplementary Fig. 7a). First, we tested that the resulting fluorescence protein had the predicted molecular weight and was well recognized by JMJD3 antibody when ectopically expressed (Supplementary Fig. 7a, b). Next, we expressed mEGFP-JMJD3 in HEK293T cells and performed an in vitro droplets assay using nuclear extracts. Our data shows that the mEGFP–JMJD3 protein forms droplets that do not appear when we overexpress mEGFP alone, reflecting that the droplets can be attributed to JMJD3, and not to the mEGFP tag (Fig. 5a). The droplets showed features [circularity, convexity and aspect ratio] that are characteristic of a liquid-like nature (Fig. 5a, bottom panels). Interestingly, some of these in vitro droplets were shared with MED15, a well-known component of enhancers that forms nuclear condensates (Supplementary Fig. 7c, d). When we overexpressed mEGFP-JMJD3, mCherry-MED15 or both we observed that 57% of JMJD3 droplets colocalized with MED15 (Supplementary Fig. 7d).

In fixed cells, overexpression of mEGFP–JMJD3 formed nuclear puncta (Fig. 5b), and the intensity of these puncta increased along with the amount of JMJD3 inside the cell (Fig. 5b); in fact, at 0.25 ug of plasmid overexpressed, we started to observe aggregates (see below). Next, we tested the sensitivity of these condensates to the aliphatic alcohol 1,6-hexanediol, a chemical compound that has been demonstrated to disrupt the hydrophobic interactions that sustain the phase-separated droplets^57^. We observed that the treatment led to a reduction in the number and size of JMJD3 puncta (Fig. 5c). Importantly, endogenous JMJD3 also formed nuclear puncta as detected by immunofluorescence using two different antibodies against JMJD3 in NSCs and HEK293T cells (Fig. 5b, without transfection, d and Supplementary Fig. 8a), ruling out the possibility that the observed puncta could be an overexpression artifact. These data suggest that JMJD3 condensates occur at endogenous levels and that these condensates represent a separated phase inside the cell.

Liquid-like condensates have been suggested to exhibit a remarkable dynamic nature, and their internal molecules have been described to diffuse rapidly^12,58^. Based on this, we sought to analyze whether the JMJD3 puncta exhibited liquid-like properties by analyzing the rate of fluorescence recovery after photobleaching (FRAP) of the overexpressed mEGFP-JMJD3^9,59^. After photobleaching mEGFP-JMJD3 puncta recovered fluorescence almost completely on a time scale of seconds (Fig. 5e, Supplementary Fig. 8b and Supplementary Movie 1), in agreement with what is observed for other proteins that form either liquid-like condensates (BRD4 and MED1) or membrane-less organelles^60^. We also calculated the mobile fraction (the molecular pool that undergo exchange within the FRAP zone), which corresponds to 0.95 for this protein. Moreover, the aggregates that appear when high levels of proteins are overexpressed (mentioned above) showed reduced mobility in FRAP assays (Supplementary Movie 2). These data suggest that JMJD3 droplets exhibit liquid-like properties and that its conforming molecules exchange rapidly between the condensates and the surrounding.

### JMJD3 IDR is necessary for condensate formation

**Fig. 6:**
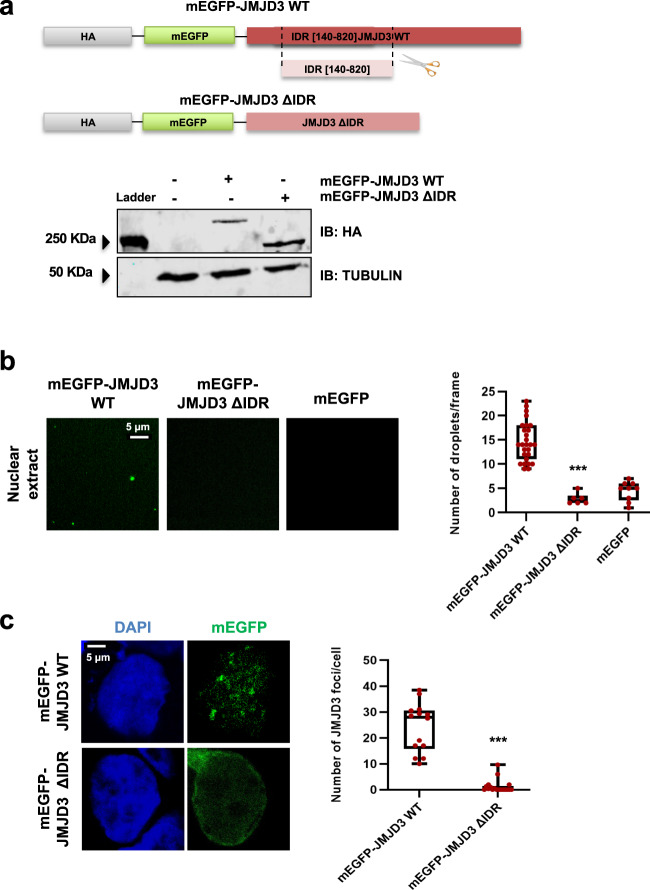
JMJD3 IDR is essential for condensate formation. **a** mEGFP–JMJD3 and mEGFP–JMJD3 ΔIDR expression vectors were transfected into HEK293T (0.05ug). 24 h later total protein extracts were prepared and the JMJD3 (HA) and TUBULIN levels were determined by immunoblot. The image shown is representative of two independent experiments. Source data are provided as a Source Data file. **b** mEGFP, mEGFP–JMJD3 and mEGFP-JMJD3 ΔIDR proteins were analyzed using droplet-formation assays in nuclear extracts at room temperature in the presence of 150 mM NaCl. Quantifications of the droplets are displayed on the right. Data are the mean ± SEM. Boxes comprise values from Q1 to Q3 of the dataset; line corresponds to median value; whiskers show the data range (from min. to max. values within dataset). ***p < 0.001 (*P* values were calculated using one-tailed Student’s *t* test, *p* = 0.000113231). Droplets in 5 fields in each group from three biologically independent experiments were quantified. Scale bar, 5 μm. Source data are provided as a Source Data file. **c** Confocal microscopy images of HEK293T cells transfected with 0.05 ug mEGFP-JMJD3 or mEGFP-JMJD3 ΔIDR. The images are representative of three biologically independent experiments. Quantifications of the number of JMJD3 puncta are shown on the right. Data show the mean ± SEM. Boxes comprise values from Q1 to Q3 of the dataset; line corresponds to median value; whiskers show the data range (from min. to max. values within dataset). ***p < 0.001 (*P* values were calculated using one-tailed Student’s *t* test, *p* = 2.5178E-09). *n* = 20 transfected cells in each group were quantified. Scale bar, 5 μm. Source data are provided as a Source Data file.

As the condensation of molecules into liquid-like droplets has been related to the IDRs present in the conforming proteins^61^, we chose to investigate whether the proline-rich IDR of JMJD3 is necessary for being part of biological condensates. To do this, we deleted the proline-rich IDR domain (amino acids 140–820) from our previously characterized mEGFP-JMJD3 plasmid, and we named this new construct mEGFP-JMJD3 ΔIDR (Fig. 6a). We ectopically expressed this protein (Fig. 6a) and analyzed its ability to form droplets in nuclear extracts. The results in Fig. 6b demonstrate that the mEGFP-JMJD3 ΔIDR protein did not form droplets in vitro. We next investigated the competence of mEGFP-JMJD3 ΔIDR to form puncta in fixed cells. These two versions of the protein were distributed between nucleus and cytoplasm, even though the JMJD3 ΔIDR shows some bias for the cytoplasm. Our immunofluorescence experiments revealed that the JMJD3 mutant was unable to form puncta (Fig. 6c). These data support that the IDR domain and probably the prolines are necessary for JMJD3 phase separation. Even though we acknowledge that identifying the precise amino acids involved in the condensation would benefit our work, both the length and the complexity of the IDR domain prevented this analysis. Nonetheless, our data suggest that not only one type of amino acid but also several contribute to JMJD3 phase separation.

Once known the relevance of the IDR domain, we analyzed if the catalytic activity of JMJD3 plays a role in the formation of condensates. To do that, we used a plasmid encoding JMJD3 mutated at the catalytic domain (JMJD3 HE > AA) (Supplementary Fig. 9a). This mutant lacks the capability of demethylating the H3K27me3 mark and functions as a dominant negative form of JMJD3^23^. Experiments in fixed cells revealed that the JMJD3 HE > AA mutant formed nuclear puncta of the same intensity and volume than those of the wild type version of JMJD3, suggesting that the catalytic activity is not essential for condensate formation (Supplementary Fig. 9a, c). To further prove that the catalytic activity of JMJD3 is not required for condensate formation, we employed a specific inhibitor of the JMJD3 catalytic activity named GSK-J4^62^. We treated the cells for 6 h, a period of time that was enough to effectively inhibit JMJD3 (Supplementary Fig. 9b). Using the GSK-J4 inhibitor, we observed puncta formation of the same intensity and volume than those of the non-treated cells (Supplementary Fig. 9b, c). Moreover, the overexpression of JMJD3 HE > AA led to the same *Chst8* transcriptional activation as the overexpression of JMJD3 WT in HEK293T cells, where TGFβ is active (Supplementary Fig. 9d). Altogether our data illustrate that the catalytic activity of JMJD3 is not required for either condensate formation or *Chst8* full transcriptional activation.

### JMJD3 promotes gene transcription and enhancer-cluster assembly

**Fig. 7:**
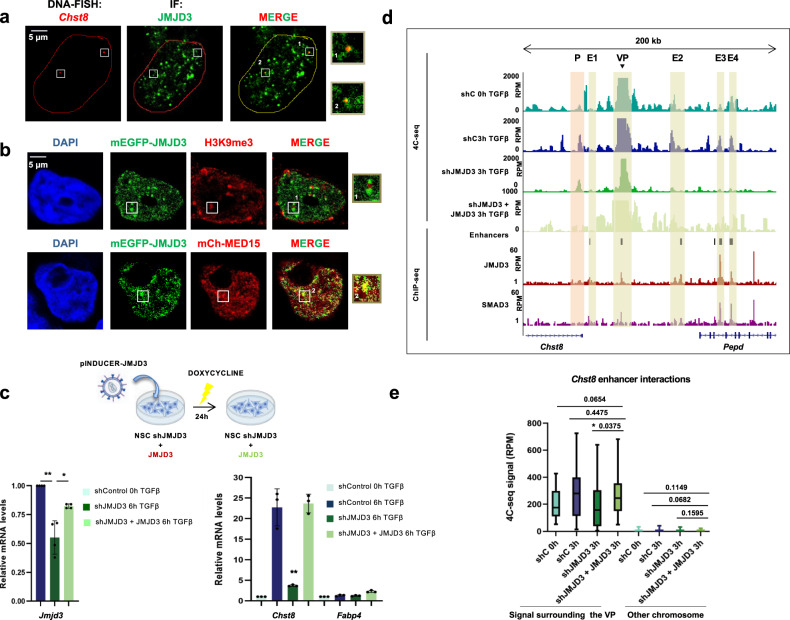
JMJD3 promotes *Chst8* gene transcription and enhancer-cluster assembly. **a** Immuno-FISH for JMJD3 protein (green) and *Chst8* locus (red) on HEK293T cells. *Chst8* FISH signal colocalizes with JMJD3 condensates (on the right). Results are representative of three independent experiments. **b** In HEK293T cells expressing mEGFP-JMJD3 (transfection of 0.05 ug, green) the localization of the H3K9me3 mark and MED15 was analyzed using immunofluorescence staining with an anti-H3K9me3 antibody (red). MED15 localization was analyzed following the red signal in cells co-expressing mEGFP-JMJD3 and mCherry-MED15. Nuclei were visualized with DAPI (blue). Colocalizations are shown in yellow. Scale bar, 5 μm. The images are representative of three independent experiments with similar results**. c** JMJD3-depleted NSCs (shJMJD3) expressing JMJD3 (shJMJD3 + JMJD3) were treated with TGFβ for 6 h. Total RNA was prepared and the mRNA expression levels of the *Chst8* gene were determined by qPCR. mRNA levels of the *Fapb4* gene were used as a negative control. Values were normalized to the *Gapdh* gene. Data are presented as mean values +/− SEM. *p < 0.05, **p < 0.01 (*P* values were calculated using two-tailed Student’s *t* test, *p* = 0.00850428 and *p* = 0.03608055 (*Jmjd3*); *p* = 0.00971346 (*Chst8*)). Results are representative of four independent experiments. Source data are provided as a Source Data file. **d** UCSC Genome Browser captures show 4C-seq profiles spanning 200 kb around the VP *Chst8* enhancer (black arrow) in control, shJMJD3 and shJMJD3 + JMJD3 NSCs treated with TGFβ during 3 h. ChIP-seq signals of SMAD3 and JMJD3 upon TGFβ stimulation are shown. The location of the members of the EC is also indicated with yellow boxes. **e** Boxplot displays the averaged values obtained from two biological independent replicates of RPM signals of the peaks located 500 kb around the VP enhancer - excluding the nearest ±20 kb - (mm10 chr7:33841896-35860773) in control, shJMJD3 and shJMJD3 + JMJD3 NSCs treated with TGFβ during 3 h. An independent region located in another chromosome (mm10 chr4:33076383-35216108) was used as a negative control. Boxes comprise values from Q1 to Q3 of the dataset; line corresponds to median value; whiskers show the data range (from min. to max. values within dataset). p-values are the result of a Wilcoxon-Mann–Whitney test.

As our data indicates that JMJD3 can form nuclear condensates, which have been widely related to transcription^8,63–65^, we hypothesized that this ability could be contributing to the JMJD3-mediated transcriptional activity on the *Chst8* locus. To test this idea, we co-imaged the *Chst8* locus and JMJD3 foci by performing an immuno-FISH experiment. A clear colocalization of JMJD3 condensates with the *Chst8* locus was detected (Fig. 7a). We also observed that JMJD3 nuclear condensates are excluded from regions marked by the repressive mark H3K9me3 (Fig. 7b), conversely, active sites of transcription marked by MED15 colocalize with JMJD3 condensates (Fig. 7b).

Finally, to conclude that JMJD3-mediated contacts indeed contribute to *Chst8* transcription, we stably integrated in the shJMJD3 NSCs a construct that, upon doxycycline treatment, overexpresses JMJD3 (Fig. 7c top and methods). After 24 h of induction, these cells displayed JMJD3 expression levels similar to the endogenous levels (Fig. 7c, left). In these conditions, we measured the expression of *Chst8* and we performed 4C-seq experiments to analyze the genomic contacts in response to TGFβ. The right panel on Fig. 7c shows a full rescue of *Chst8* transcription upon induction of JMJD3 in the shJMJD3 NSCs without affecting *Fabp4*, a negative control. As hypothesized, after reintroduction of JMJD3, the genomic contacts were recovered (Fig. 7d, e), in particular, contacts between the *Chst8 EC* were efficiently restored (Fig. 7d).

Altogether, these results support a model in which JMJD3 facilitates the assembly of the described *Chst8* EC, probably through the formation of IDR-driven phase-separated condensates enriched in key factors that enable gene expression.

## Discussion

In this work, we provide a molecular description of an enhancer cluster formation in response to the TGFβ signaling pathway during neurogenesis. Our data uncover an unforeseen role of TGFβ reorganizing the chromatin fiber in a JMJD3 histone demethylase-dependent manner. JMJD3 promotes the establishment of enhancer–enhancer and enhancer–promoter contacts that ultimately modulate *Chst8* enhancer activity, and thus the NSCs gene expression program.

Mammalian promoters are normally surrounded and regulated by multiple enhancers. Enhancer–enhancer contacts have been described in the literature (for review^66^) and demonstrated by chromatin conformation capture techniques, and cell imaging^67,68^. Nonetheless, the contribution of individual enhancers to enhancer clusters and their impact on target gene regulation is still an open debate. Our results show that upon TGFβ treatment, the *Chst8* locus is reorganized in the 3D space (Fig. 2a, b), bringing into proximity cis-regulatory regions to facilitate an accurate *Chst8* transcriptional response (Fig. 2c, e). Interestingly, an allelic variant of a *Chst8* exon has been involved in a peeling skin syndrome (OMIM #616265), highlighting the importance of a controlled *Chst8* gene response^69^. Even though we have focused on the *Chst8* locus, we have also demonstrated by 4C-seq assays, that TGFβ reorganizes other loci entailing TGFβ-responsive genes in a similar manner (Supplementary Fig. 3). Altogether, these results highlight the essential contribution of the TGFβ pathway as a major force driving the 3D organization of the chromatin.

In this work, we have provided an answer to an open question from our previous work^33^: why is JMJD3 required at enhancers that are not marked by H3K27me3? Here, we reveal a novel function for JMJD3 mediating the TGFβ-driven enhancer–promoter and enhancer–enhancer contacts. This role agrees with previous literature that elegantly demonstrated that JMJD3 facilitates enhancer–promoter looping during endoderm differentiation^70^. How can JMJD3 facilitate TGFβ-driven transcriptional response through 3D-chromatin organization? Past work in our laboratory demonstrated that JMJD3 is required for TGFβ-driven activation at different levels: promoter, enhancer, and gene body (see below); additionally, some functions were demonstrated to be dependent on the demethylase catalytic activity, whereas others were not^23,33^. Here, we provide a further molecular explanation for this contribution, unveiling the previously unknown JMJD3 IDR as a crucial protein region that enables the formation of biomolecular condensates (Fig. 6). Interestingly, recent data demonstrated that UTX, another member of the same family of KDMs, undergoes LLPS driven by an IDR domain. In agreement with our work, Shi et al. have demonstrated that the IDR facilitates higher-order chromatin interactions and mediates tumor suppression in a catalytic independent manner^71^. With our study, we propose that JMJD3 phase separates in NSCs by establishing multivalent interactions through its IDR and that this condensation might be an important mechanism to enable a precise TGFβ-response. Indeed, recent studies have suggested a model involving condensate formation to explain both the initiation and the elongation transcriptional stages^8,63–65^. Following this model, initiation requires the condensate assembly of Mediator, transcription factors, coactivators and non-phosphorylated RNAPII. The second step consists in an elongation condensate arrangement that includes phosphorylated RNAPII, RNA processing and elongating factors, and RNA itself. Once at the end of the gene, hypophosphorylated CTD is released from the condensates so that it can be re-incorporated into the initiation condensates^63,72^. Having this model in mind, we speculate that JMJD3 could be participating in both the initiation and the elongation condensates. JMJD3 is found at promoters and enhancers in response to TGFβ where it interacts with SMAD3. This transcription factor also undergoes LLPS through its IDR^73^. Previous data from the laboratory^23^ demonstrated that SMAD3 interacts with JMJD3 through its linker region, which contains an IDR, suggesting that SMAD3 and JMJD3 IDRs may be engaging in multivalent interactions. Interestingly, the contacting genomic regions in the *Chst8* ECs were bound by JMJD3 and SMAD3, suggesting that both factors may contribute to the formation of the initiation condensates required for transcription activation. Moreover, it has been suggested that phase separated condensates are formed at EC^10,11,74,75^. In agreement, our data indicates that JMJD3 contributes to condensates that take place at EC potentially together with the enhancer machinery (e.g. general factors such as Mediator, RNAPII, or TGFβ pathway-specific factors such as SMAD3 or CHD8). On the other hand, our lab and others have previously demonstrated that JMJD3 interacts with the elongating form of the RNAPII^36^ and with elongation factors^42^ and that it is essential for the elongation stage^36,42^. Thus, we speculate that JMJD3 could favor elongation by promoting or forming part of the elongating condensates.

Based on our results, we hypothesize that JMJD3 condensates could facilitate transcription in different ways. JMJD3 might concentrate SMAD3, CHD8, and MED15, and the general elongation factors in a compartment to make transcription kinetically more efficient as it has been proposed for other factors^11,61,63^. Alternatively, it might physically insulate TGFβ-responsive transcriptional machinery from its regulators to prevent inactivation (e.g., insulating the active phospho-SMAD3 from phosphatases). Finally, it is conceivable that the JMJD3-mediated chromatin 3D organization could be a critical determinant that allows biomolecular condensate formation at active genome loci. The enhancer-enhancer and enhancer-promoter contacts that contain large amount of transcription factor binding sites might work as a required nucleation step for the biomolecular condensates.

In these or other scenarios, as TGFβ is an essential regulator of cell proliferation, survival, differentiation, and plays a critical role in cancer and neurodegenerative disorders^76^, understanding how the phase separation of its coactivator JMJD3 impacts its regulatory processes could undoubtedly contribute to understanding the crosstalk between diseases and development, and potentially provide new therapeutic targets.

## Methods

### Cell culture and cell treatments

Briefly, mouse NSCs were dissected from cerebral cortices of C57BL/6 J mouse fetal brains (E12.5) and cultured in poly-D-lysine (5 μg/ml, 2 h 37 °C) and laminin (5 μg/ml 37 °C, 4 h 37 °C) precoated dishes^77^ and have subsequently been maintained in culture as a stable cell line. NSCs were grown with a medium prepared by mixing equal parts of DMEM F12 (without Phenol Red, Gibco) and Neural Basal Media (Gibco), Glutamax (1%), N2 and B27 supplements (Gibco), sodium pyruvate (1 mM), non-essential amino acids (0.1 mM), Heparin (2 mg/l), Hepes (5 mM), bovine serum albumin (25 mg/l) and β-mercaptoethanol (0.01 mM)^23^. Fresh recombinant human Epidermal Growth Factor (EGF) (R&D systems) and Fibroblast Growth Factor (FGF) (Invitrogen) to 20 ng/ml and 10 ng/ml final concentrations respectively were added to the media. TGFβ (Millipore) was used at a final concentration of 5 ng/ml. Human HEK293T cells were cultured in DMEM supplemented with 10% of fetal bovine serum (Gibco) and 1% of Penicillin/Streptomycin^78^.

### Antibodies and reagents

Antibodies used were anti: JMJD3^28^ (raised in the laboratory using amino acids 798–1095, dilution 1:200 for immunofluorescence (IF), 1:1000 for western blot (WB); and Abcam, ab38113, dilution 1:250 for IF), DAPI (ThermoFisher, D1306, dilution 1:500 for IF), β-TUBULIN (Millipore, MAB3408, dilution 1:5000 for WB), HA tag (Abcam, ab20084, dilution 1:5000 for WB), H3K9me3 (Abcam, ab8898, dilution 1:250 for IF), H3K27me3 (Millipore, 07449, dilution 1:500) and Goat anti-Rabbit IgG (H + L) Highly Cross-Adsorbed Secondary Antibody, Alexa Fluor™ Plus 488 (Invitrogen, A32731, dilution 1:1000 for IF), Goat anti-Mouse IgG (H + L) Highly Cross-Adsorbed Secondary Antibody, Alexa Fluor™ Plus 555 (Invitrogen, A32727, dilution 1:1000 for IF). TGFβ and doxycycline hyclate were acquired from Millipore (GF111 and 324385 respectively). The doxycycline was used at a concentration of 1 ug/ml for 24 h. GSK-J4 inhibitor was acquired from Selleckchem (GSKJ4 HCl S7070) and used at a concentration of 1 uM for 6 h.

### Plasmids

Specific lentiviral vectors were purchased from Sigma: pLKO.1-random (CAACAAGATGAAGAGCACC), pLKO.1-shSMAD3 (CCTTACCACTATCAGAGAGTA), and pLKO.1-shJMJD3 (CCTCTGTTCTTGAGGGACAAA). mEGFP was amplified by PCR from the pCMV-mEGFP-C1 vector and cloned into the pCMV-HA-JMJD3 plasmid by using an Acc65I restriction site. Primer sequences are described in Supplementary Table 1. pCMV-HA-mEGFP-JMJD3 ΔIDR (140-820) was obtained by digesting pCMV-HA-mEGFP-JMJD3 vector with HindIII enzyme and self-ligating the resulting fragment. pCMV-HA-mEGFP-JMJD3 HE > AA (H1390A/E1392A) was obtained by replacing JMJD3 from pCMV-HA-mEGFP-JMJD3 vector with JMJD3 HE > AA (H1390A/E1392A). This mutant version was cut from pCIG-JMJD3-H1390A/E1392A vector with XhoI and XagI enzymes. pInducer-JMJD3 WT and pInducer-JMJD3 HE > AA (H1390A/E1392A) vectors were obtained through an LR recombination reaction taking advantage of the Gateway Recombination Cloning Technology.

### Lentiviral transduction

Lentiviral transduction to generate the knockdown cell lines was carried out as previously described^79^. Briefly, lentiviral particles are produced in HEK293T cells by cotransfecting plasmids encoding the shRNA together with pCMV-VSVG and pCMV-GAG-POL plasmids. After 24 h, supernatants containing lentiviral particles are collected and centrifuged in a sucrose bed at 57000 x *g* for 2 h. Then, viral particles are resuspended in NSCs medium, and they are directly used for NSCs infection. One day after infection, cells are selected with puromycin (Sigma #P8833) at a concentration of 2 ug/ml for several days.

### 4C-seq assay

The 4C-seq experimental protocol was based on^80,81^. Briefly, 12 × 10^6^ mNSCs were fixed for 30 min using 1% of formaldehyde at room temperature. The fixing reaction was quenched with glycine 0.125 M for 10 min. After 2 washes with PBS, cell pellets were resuspended in 5 ml of cytoplasmic lysis buffer (50 mM Tris–HCl pH 7.5, 150 mM NaCl, 5 mM EDTA, 0.5% NP-40, 1% Triton X-100 and protease inhibitors) during 10 min on ice. Lysates were centrifuged for 5 min at 650 x *g* and 4 °C. Nuclei were resuspended in 0.5 ml of NlaIII buffer with 0.3% SDS and they were incubated at 37 °C and 900 rpm for one hour. After that, Triton X-100 was added to a final concentration of 2% followed by 1 h of incubation at 37 °C and 900 rpm. Next, DNA was digested overnight at 37 °C and 900 rpm with 400 U of NlaIII, which was afterward inactivated by adding SDS to a final concentration of 1.6% and incubating for 20 min at 65 °C and 900 rpm. The digested chromatin was transferred to 50 ml tubes and 6.125 ml of 1.15X ligation buffer (50 mM Tris-HCl pH 7.6, 10 mM MgCl_2_, 1 mM ATP, 1 mM DTT) and 1% of Triton X-100 were added and incubated during 1 h at 37 °C and 1000 rpm. Digested chromatin was ligated with 100 U of T4 DNA ligase for 8 h at 16 °C and then, treated with RNase A 1 mg/ml for 45 min at 37 °C. Decrosslinking step was performed by adding 1 mg/ml of proteinase K and incubating at 65 °C overnight. DNA was purified by standard phenol-chloroform extraction followed by ethanol precipitation and resuspended in 100 ul of H_2_O. At this point, proper digestion and ligation were evaluated by visualizing the DNA in an agarose gel. A second digestion with 50 U of DpnII was performed at 37 °C overnight. Enzyme and buffer were removed from the sample by phenol-chloroform extraction followed by ethanol precipitation and once purified, DNA samples were resuspended in 500 ul of H_2_O. A second ligation was carried out by adding 200 U of T4 DNA ligase in a final volume of 14 ml of 1X ligation buffer. The mixture is incubated overnight at 16 °C and after the last round of phenol-chloroform extraction and ethanol precipitation, the DNA was resuspended in 100 ul of H_2_O and purified with a Qiagen PCR purification column. The efficiencies of the second digestion and ligation are tested by DNA electrophoresis, when correct, this sample comprises the 4C-library sample. Using the indicated viewpoints, inverse PCR reactions were performed using the Expand Long template PCR system (Roche #11681834001) with the following cycling conditions: 94 °C 2 min, 29 cycles of 94 °C 10 s – 55 °C 1 min – 68 °C 3 min and 68 °C 5 min (for *Chst8* and *Ldlrad4*) and 94 °C 2 min, 31 cycles of 94 °C 10 s – 55 °C 1 min – 68 °C 3 min and 68 °C 5 min (for *Aopep*). Primer sequences are described in Supplementary Table 2. The products of these reactions were sent for sequencing to the Erasmus Center for Biomics in Rotterdam, in an Illumina HiSeq2500 sequencer with a read depth of 100 bp in the case of the *Chst8* VP and 74 bp in the other samples. 4C-seq data have been deposited in the GEO database under the accession GSE197013 (GSE197010 for *Chst8*; GSE197011 for *Aopep*; GSE197012 for *Ldlrad4*).

### Computational analysis of the 4C-seq experiment

The obtained sequencing reads were processed using the 4C-seq pipeline named pipe4C^35^ using default parameters except for the trimLength that was set up to 36 bp, and the genome version for mapping that was *Mus musculus* mm10. Further statistical analysis was performed with R3Cseq^82^, a Bioconductor package that allows the identification of interacting genomic regions and the comparison between multiple replicates and experimental conditions.

### RNA extraction and qPCR

RNA was extracted using TRIZOL reagent (Invitrogen), following the manufacturer instructions. Reverse transcription was performed with 200–1000 ng of RNA using High Capacity cDNA reverse transcription kit (Invitrogen). qPCR was carried out with SYBR Green (Roche) in a QuantStudio 5 Real-Time PCR system (ThermoFisher Scientific) using specific primer pairs (see Supplementary Table 1).

### ChIP assays

Chromatin immunoprecipitation (ChIP) assays were performed as previously described^83^ with modifications: 6 ×10^6^ NSCs were fixed with formaldehyde 1% during 10 min. Fixation was stopped by the addition of 0.125 M of glycine. Cells were lysed in 1% SDS lysis buffer (1% SDS; 10 mM EDTA pH 8.0; 50 mM Tris–HCl pH 8.1). A sonication step to fragment the chromatin was performed in a Bioruptor sonicator (Diagenode) and shredded chromatin was used for each immunoprecipitation using the H3K27me3 antibody. Magna ChIP Protein A Magnetic Beads (Millipore) were used to capture the immunocomplex. DNA was purified by phenol-chloroform followed by ethanol precipitation and analyzed by qPCR with SYBR Green (Roche) in a QuantStudio 5 Real-Time PCR system (ThermoFisher Scientific) using specific primers (see Supplementary Table 1).

### ChIP-seq data acquisition

ChIP-seq data were downloaded from Gene Expression Omnibus (https://www.ncbi.nlm.nih.gov/geo/) (Accessions used in this paper are specified in Supplementary Table 3). ChIP-seq captions were obtained from UCSC genome browser^84,85^.

### Western blot

Immunoblotting was performed using standard procedures. An ECL kit (Amersham) was used to visualize the results. Uncropped and unprocessed scans of the performed Western Blots are provided in the Source Data file.

### CRISPR-Cas9

In order to delete the *Chst8* viewpoint (VP) enhancer, primer pairs of gRNA (Supplementary Table 1) were designed flanking the mm10 coordinates chr7:34846279-34849157 using the online tool http://crispr.mit.edu/. Selected primer pairs have an off-target score of 80 (left) and 90 (right) and an on-target score of 69 (left) and 65 (right). gRNAs were cloned into the pX330-U6-Chimeric_BB-CBh-hSpCas9 vector (Addgene #42230) using BbsI sites. Plasmids were nucleofected in NSCs with an Amaxa Nucleofector (Lonza) following manufacturer instructions. After puromycin selection (0.8 ug/ml) and detection analysis with conventional PCR, heterogeneous population carrying a majority of homozygotic deletions was used for experiments.

### Droplet assays in nuclear extracts

5 ug of vector encoding *Jmjd3* cDNA fused to mEGFP were transfected into 20 × 10^6^ HEK293T cells as previously described^86^. Nuclear extracts were prepared at a concentration of 2 mg/ml and they were used for droplet formation assays by diluting them 1:1 with buffer (10% glycerol, 20 mM HEPES). The final droplet buffer conditions were 20 mM HEPES, 150 mM NaCl, 15% glycerol, 3.75 mM EGTA, 2.5 mM MgCl2, and 1.25 mM CaCl2. The reactions were incubated for 30 min and loaded onto a glass-bottom 384-well plate (Cellvis P384-1.5H-N) 5 min before imaging on an Automated Inverted Microscope Leica Thunder 3D Live Cell using a 63x water immersion objective (NA = 1.2).

### Quantification of droplets liquid-like features

Droplets shape descriptors “circularity” and “aspect ratio” were quantified using the “Analyze particles” plugin in Fiji; “convexity” was calculated running the “Calculate Convexity and Solidarity” macro in Fiji^12,87,88^. Each image was cropped, and a threshold was set so that each droplet could be seen as an individual object. The results showed in the figure correspond to the measurement of 150 droplets.

### Fluorescence recovery after photobleaching (FRAP) in live cells

HEK293T cells were transfected with 0.05 ug of mEGFP-JMJD3 vector and grown on glass dishes coated with 5 ug/ml of poly-D-lysine in 1.5 ml of DMEM media as previously described in this manuscript. FRAP was performed on a Zeiss LSM780 confocal microscope equipped with a 40x water immersion objective (NA = 1.2) and a GaAsP photomultiplier detector. Acquisition settings were optimized for fast imaging and low photobleaching, using 488 nm laser excitation power of 0.15% (AOTF), a detector gain of 780, a pixel dwell of 1.27 usec and a pixel size of 140 nm. Bleaching was performed after 5 previous images by using 488 nm laser excitation power of 100% (AOTF), a pixel dwell of 2.55 and 10 iterations, over a 6 × 6 pixels region of interest (ROI) focused on the interest spot. Acquisition was set to intervals of 1 s for both the pre-bleach imaging and the post-bleach recovery time.

Intensity recovery quantification was performed using Fiji^89^. A macro was programmed that (i) registered the whole time-lapse to avoid live cell fluctuations; (ii) allowed the user to draw a ROI around the bleached spot; (iii) fine-tuned the selected ROI by applying a threshold on the time projection of the signal spot; (iv) allowed the user to select a background ROI; (v) automatically segmented the target nucleus and created a ROI where to calculate the bleaching gap and the bleach depth; (vi) automatically measured the intensity of the three ROIs over the time-lapse and (vii) delivered the data in a *.txt format. The code and further details can be downloaded from https://github.com/MolecularImagingPlatformIBMB. For each cell, three rounds of image quantification were collected, as to minimize the experimental error due to image analysis quantification. Statistics and fitting were performed using the easy-FRAP web^90^.

### 1,6-Hexanediol treatment for live imaging of cells

HEK293T cells were transfected with 0.05ug of HA-EGFP-JMJD3 vector and grown on glass dishes coated with 5 ug/ml of poly-D-lysine in 1.5 ml of DMEM media as previously described in this manuscript. They were imaged before treatment on a 37 °C heated stage of a Zeiss LSM780 Confocal using Zen software to establish a baseline. The Spectral (GaAsP) detector and a 40x water immersion (NA = 1.2) objective were used. After the fifth acquisition, 1,6-Hexanediol (#240117, Sigma) was added to cells at a final concentration of 6% in normal media, and images were again taken for 5 min of continuous treatment. Raw images were processed using Fiji software for posterior analysis and quantifications. Representative and consistent images of puncta disassembly at 60 and 120 s are presented.

### Focus calling (Immunofluorescence, 1,6-Hexanediol treatment)

Foci were called using the “Object Counter 3D” plugin in Fiji. For each image, the “threshold” parameter was set so that each focus could be seen as an individual object. The parameters showed (number of foci/cell, intensity, and volume) are the mean of the results obtained for each image with the “Statistics” function of the plugin (the number of cells used for the quantifications is specified in each figure legend).

### Indirect immunofluorescence

Immunostaining assays were carried out as previously described^91^. Briefly, cells were fixed with 4% paraformaldehyde for 20 min and permeabilized with PBS-Triton X-100 (0.5%). Cells were blocked for 1 h at room temperature in 0.5% BSA (in PBS with 0.1% Triton X-100) before overnight incubation at 4 °C with primary antibodies. Next, cells were incubated with Alexa-conjugated secondary IgG antibodies (Jackson ImmunoResearch) and 0.1 ng/ul DAPI (Sigma) for 2 h at room temperature. For cells overexpressing mEGFP or mCherry-fusion proteins, the intrinsic fluorescence of the molecules was captured without using either primary or secondary antibodies. Images were captured by Leica SP5 confocal microscope using LAS-AF software.

### Immunofluorescence with DNA FISH

Mouse NSCs were grown on glass dishes precoated with 5 ug/ml of poly-D-lysine and 5 ug/ml of laminin and treated with TGFβ for 3 h as previously described in this manuscript. After fixation in 4% paraformaldehyde containing 0.1% Triton X-100 solution for 10 min, cells were incubated in 10 mM glycine for 30 min and washed with PBS three times. Then, cells were dehydrated by performing sequential washes with 70%, 85% and 100% ethanol for 2 min at RT and then air dried. Probe hybridization mixture was made by mixing 8 ul of FISH Hybridization Buffer (Empire Genomics) and 2 ul of the FISH probe (see below). The 10 ul of mixture was added on a slide. Genomic DNA and probes were denatured at 75 °C for 7 min and slides were incubated at 37 °C in the dark overnight. The coverslip was removed from the slide and washed twice at 73 °C for 5 min with Wash solution 1 (0.3% NP-40/0.4x SCC) and twice at room temperature for 2 min with Wash solution 2 (0.3% NP-40/3x SCC). Then immunofluorescence was performed. Cells were fixed again in 4% paraformaldehyde for 10 min and washed with PBS three times. Permeabilization was done in 0.2% Triton X-100 in PBS for 10 min. After washing with PBS, cells were blocked at room temperature with PBG for 1 h. Then, cells were incubated with the primary antibody (anti-JMJD3 ab38113) at 4 °C overnight. After three washes with PBG, cells were incubated with the secondary antibody (Alexa anti-rabbit 488) for 45 min at room temperature. The coverslip was washed twice with PBG and twice with PBS, and nuclei were stained with DAPI for 10 min at room temperature. Coverslips were mounted with Mowiol and images were acquired on an Andor Dragonfly Spinning Disk Confocal Microscope with an oil immersion 100x (NA = 1.49) objective and a pixel size of 51 nm objective using Fusion acquisition software. Images were post-processed using Fiji^92^. FISH foci were manually identified in individual z stacks through intensity thresholds in FIJI. The DNA FISH probe was synthesized by Empire Genomics and targets the *Chst8* enhancer cluster locus (mm10 coordinates chr7:34795935-34985109).

### Sequence analysis and predictions

Protein disorder estimations were generated using three prediction algorithms, PONDR-VL3^45^, IUPred^46^ and PONDR-VSL2^47^. The predictors give a value between 0 and 1 for each amino acid, where above 0.5 is predicted to lie within a disordered region of more than 50 amino acids long. To predict the phase separation property of each protein, PSPredictor^53^ and catGRANULE^52^ predictors were used online.

Low-complexity domains presence was assessed using SEG algorithm together with MobiDB database^48^. For amino acid composition analysis, the web application Prot Pi Protein Tool https://www.protpi.ch/Calculator/ProteinTool was used. Disordered proteins were defined by the presence of a 50 residues fragment whose IUPRED median score was at least of 0.55 and that was not found in Pfam, a protein domain database. The hydrophobicity was calculated with the ExPASy website^93^ using the Hopp and Woods scale^94^ and a sliding window of 21.

### Statistical analysis

Quantitative data were expressed as mean and standard error mean (SEM) (for immunofluorescence quantifications and RNA transcription experiments). The significance of differences was assessed using the Student’s *t* test (*p < 0.05; **p < 0.01, ***p < 0.001).

### Reporting summary

Further information on research design is available in the Nature Research Reporting Summary linked to this article.

## Supplementary information

Supplementary Information

Description of additional Supplementary File

Supplementary Movie1

Supplementary Movie2

Supplementary Dataset 1

Supplementary Dataset 2

Supplementary Dataset 3

Reporting Summary

## Data Availability

The data that support this study are available from the corresponding author upon reasonable request. The 4C-seq data generated in this study have been deposited in the GEO database under accession code GSE197013. The ChIP-seq data used in this study are available in the GEO database under accession code GSM898371, GSM937827, GSE66961, GSE66961, GSE66961, GSM883646, GSE38269. Source data are provided with this paper.

## Source data

Source Data

## Code availability

The code and further details for FRAP image analysis can be downloaded from https://github.com/MolecularImagingPlatformIBMB.
