# Supplementary material for: JMJD3 intrinsically disordered region links the 3D-genome structure to TGFβ-dependent transcription activation
Source: Nat Commun. 2022 Jun 7;13:3263. doi: 10.1038/s41467-022-30614-y (PMC9174158; doi:10.1038/s41467-022-30614-y)
Supplement: Supplementary file 1 — Supplementary Information [file 41467_2022_30614_MOESM1_ESM.pdf]

JMJD3 intrinsically disordered region links the 3D-genome structure to TGFβ-dependent transcription activation

Marta Vicioso-Mantis, Raquel Fueyo, Claudia Navarro, Sara Cruz-Molina, Wilfred F.J. van Ijcken, Elena Rebollo, Álvaro Rada-Iglesias, and Marian A. Martínez-Balbás

SUPPLEMENTARY FIGURES AND TABLES

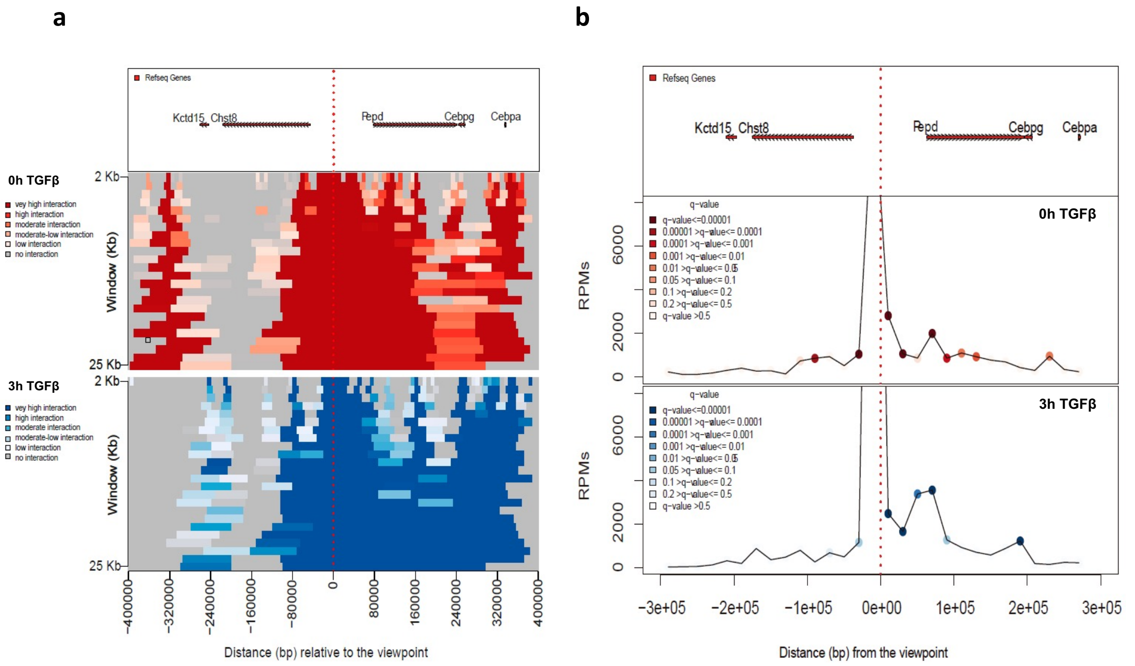

Supplementary Figure 1. **TGFβ drives the reorganization of 3D chromatin structure. a** r3Cseq domainogram shows the 3D interactions between the VP *Chst8* enhancer and the genomic regions located 400 kb either upstream or downstream. Different shades of red (Control 0 h TGFβ) or blue (Control 3 h TGFβ) illustrate the frequency of the interactions. The y-axis represents interactions identified using different window sizes. **b** r3Cseq plot showing the statistical significance of the

genomic interactions between the VP *Chst8* enhancer and the surrounding regions. Different shades of red (Control 0 h TGFβ) or blue (Control 3 h TGFβ) represent different levels of statistical significance. Plots report the results of two independent biological replicates combined.

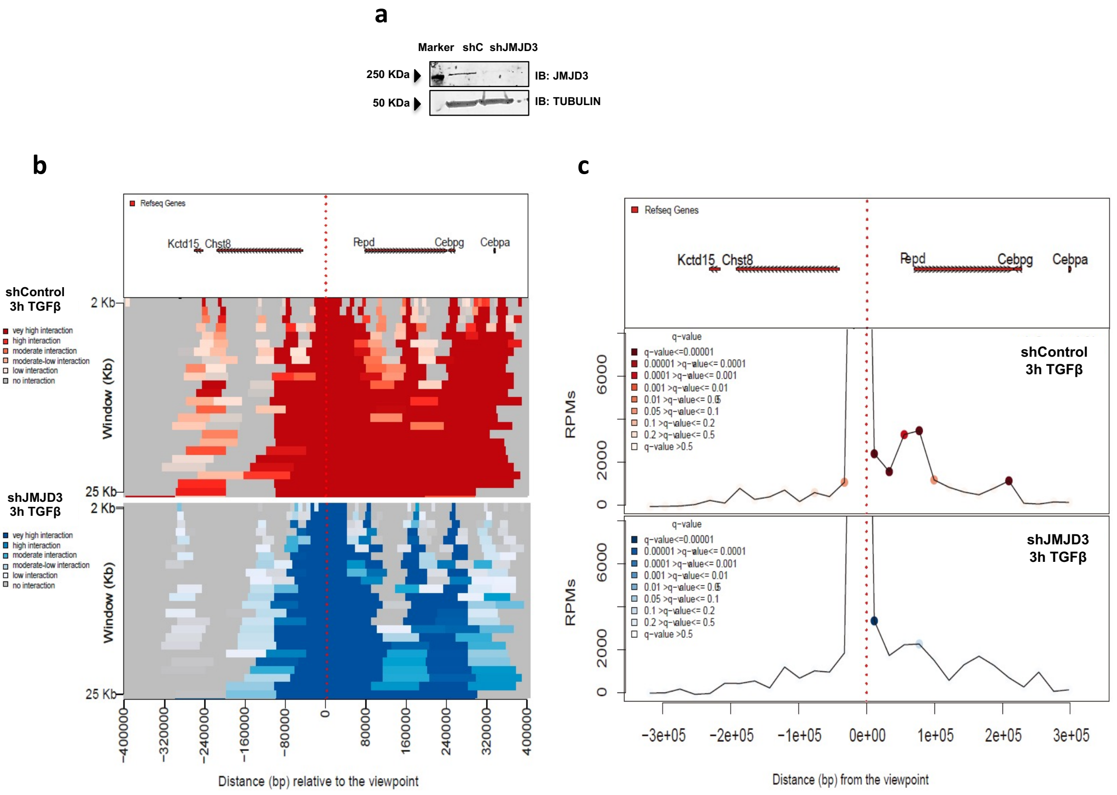

Supplementary Figure 2. **TGFβ -mediated chromatin reorganization depends on JMJD3**. **a** NSCs were infected with lentivirus expressing shRNA control (shC) or shRNA specific for JMJD3 (shJMJD3). 48 h later total protein extracts were prepared and the JMJD3 and TUBULIN levels were determined by immunoblot. The images are representatives of three independent experiments with similar results. Source data are provided as a Source Data file. **b** r3Cseq domainogram shows the 3D interactions between the VP *Chst8* enhancer and the genomic regions located 400 kb either upstream or downstream. Different shades of red (shControl 3 h TGFβ) or blue (shJMJD3 3 h TGFβ) illustrate the frequency of the interactions. The y-axis represents interactions

identified using different window sizes. **c** r3Cseq plot showing the statistical significance of the genomic interactions between the VP *Chst8* enhancer and the surrounding regions. Different shades of red (shControl 3 h TGFβ) or blue (shJMJD3 3 h TGFβ) represent different levels of statistical significance. Plots report the results of two independent biological replicates combined.

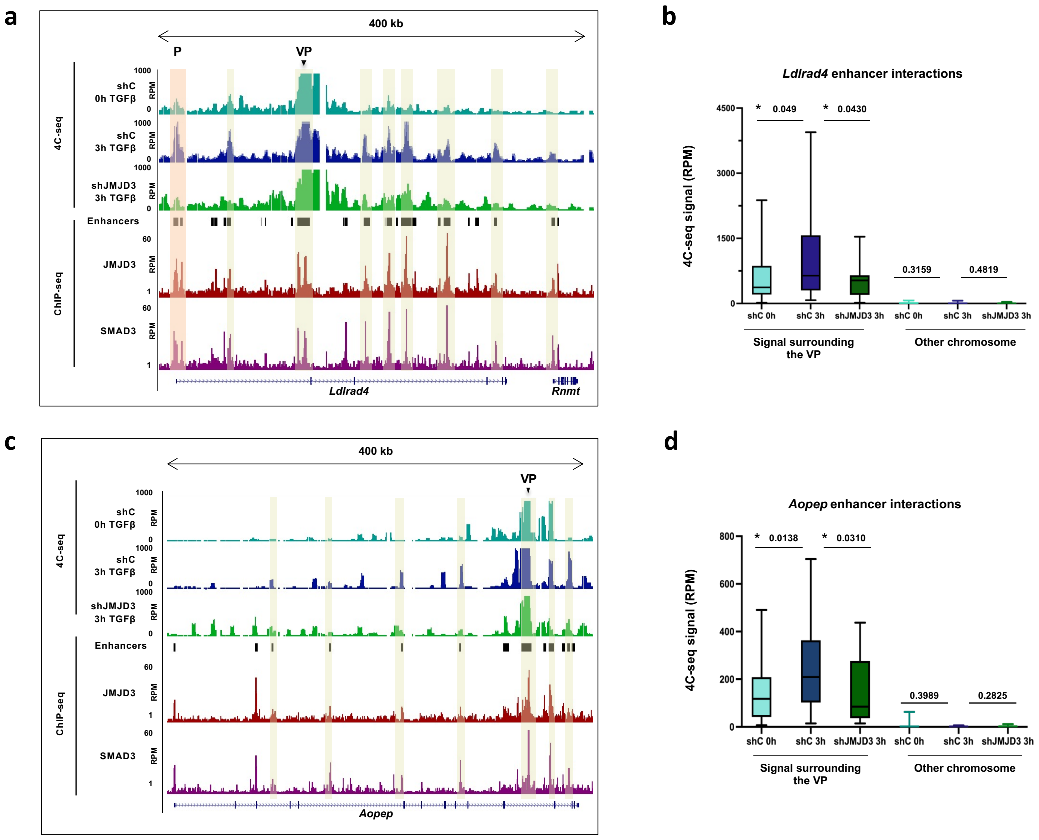

Supplementary Figure 3. **TGFβ-mediated chromatin reorganization depends on JMJD3 at *Ldlrad4* and *Aopep* loci. a, c** UCSC Genome Browser captures show 4C-seq profiles spanning 400 kb around the VP enhancers (black arrows), *Ldlrad4* (a) and *Aopep* (c) in shControl NSCs untreated or treated (3 h) with TGFβ and in shJMJD3 NSCs treated with TGFβ for 3 h. ChIP-seq signals of SMAD3 and JMJD3 upon TGFβ stimulation (0.5 and 3 h, respectively) are shown. The gene promoter is indicated with a light orange box. The location of the contacting enhancers is indicated with yellow boxes. **b, d** Boxplot displays the averaged values obtained from two biological

independent replicates of RPM signals of the peaks located 500 kb around the VP enhancers - excluding the nearest ± 20 kb -, *Ldlrad4* (b) (chr18:67,600,000-38,400,000) and *Aopep* (d) (chr13:62,680,000-63,600,000) in shControl NSCs or shJMJD3 NSCs untreated or treated with TGFβ during 3 h. An independent region located in another chromosome (chr4:33,100,000-35,180,000) was used as a negative control. Boxes comprise values from Q1 to Q3 of the dataset; line corresponds to median value; whiskers show the data range (from min. to max. values within dataset). *n*=2 biologically independent replicates were quantified. p-values are the result of a Wilcoxon-Mann-Whitney test.

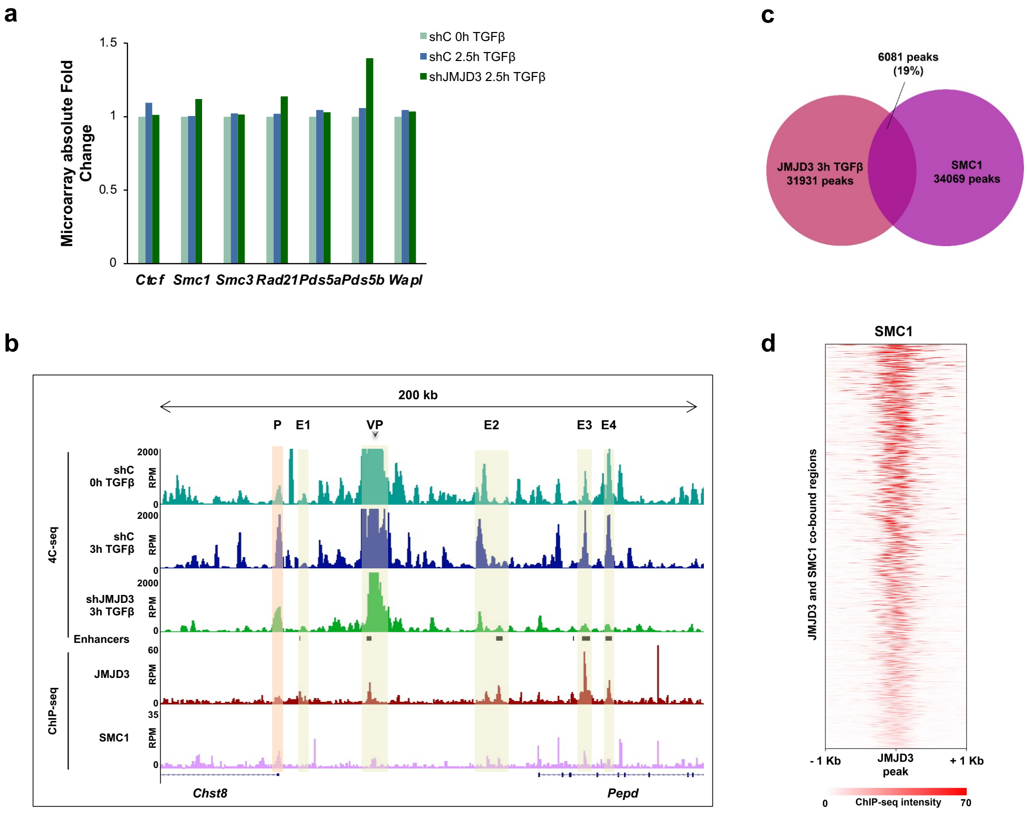

Supplementary Figure 4**. JMJD3's role mediating contacts seems to be independent of proteins involved in chromatin loop formation. a** Microarray expression data[1] showing the comparison of the expression levels of proteins involved in chromatin loop formation in NSCs shC and shJMJD3 treated with TGFβ. Data are presented as absolute

fold change values. **b** UCSC Genome Browser captures[2] showing 4C-seq results in control (shC) or JMJD3 depleted (shJMJD3) NSCs upon TGFβ treatment (3 h). ChIP-seq signal of JMJD3 and SMC1 are also depicted. Our regions of interest were JMJD3 and SMC1 signals colocalize are highlighted in light orange and yellow. The positions of enhancers are also indicated. **c** Venn diagram showing the colocalization of peaks corresponding to JMJD3 and SMC1 proteins in NSCs. **d** Heatmap showing the colocalization of SMC1 and JMJD3 at their co-bound regions.

a

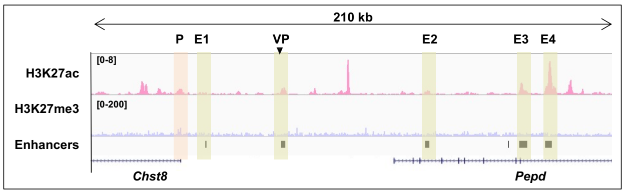

b

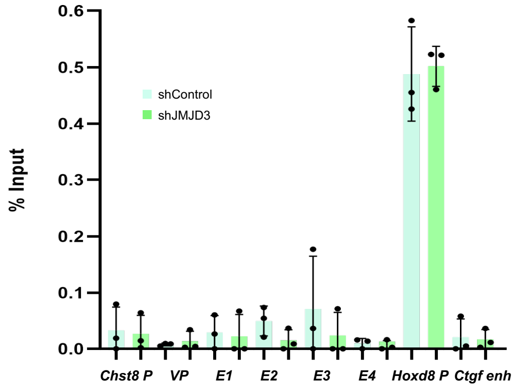

Supplementary Figure 5**. The *Chst8* locus displays low levels of H3K27me3 and these are not dependent on JMJD3. a** IGV captures showing the H3K27me3 and H3K27ac levels around the *Chst8* gene promoter and the *Chst8* EC enhancer in untreated NSCs. Tracks display H3K27me3 and H3K27ac ChIP-seq data. The positions of enhancers (defined in[3]) are also displayed. Promoter and EC are shaded in light orange and yellow respectively. **b** The levels of H3K27me3 in control and JMJD3 depleted NSCs were

determined by ChIP-qPCR at the *Chst8* promoter and enhancers forming part of the EC. *Hoxd8P*, a highly H3K27 trimethylated promoter and *Ctcf*, a non-trimethylated enhancer, were used as positive and negative controls respectively. qPCR data were normalized to the input, the IgG values were subtracted. Data are the mean ± SEM. Source data are provided as a Source Data file.

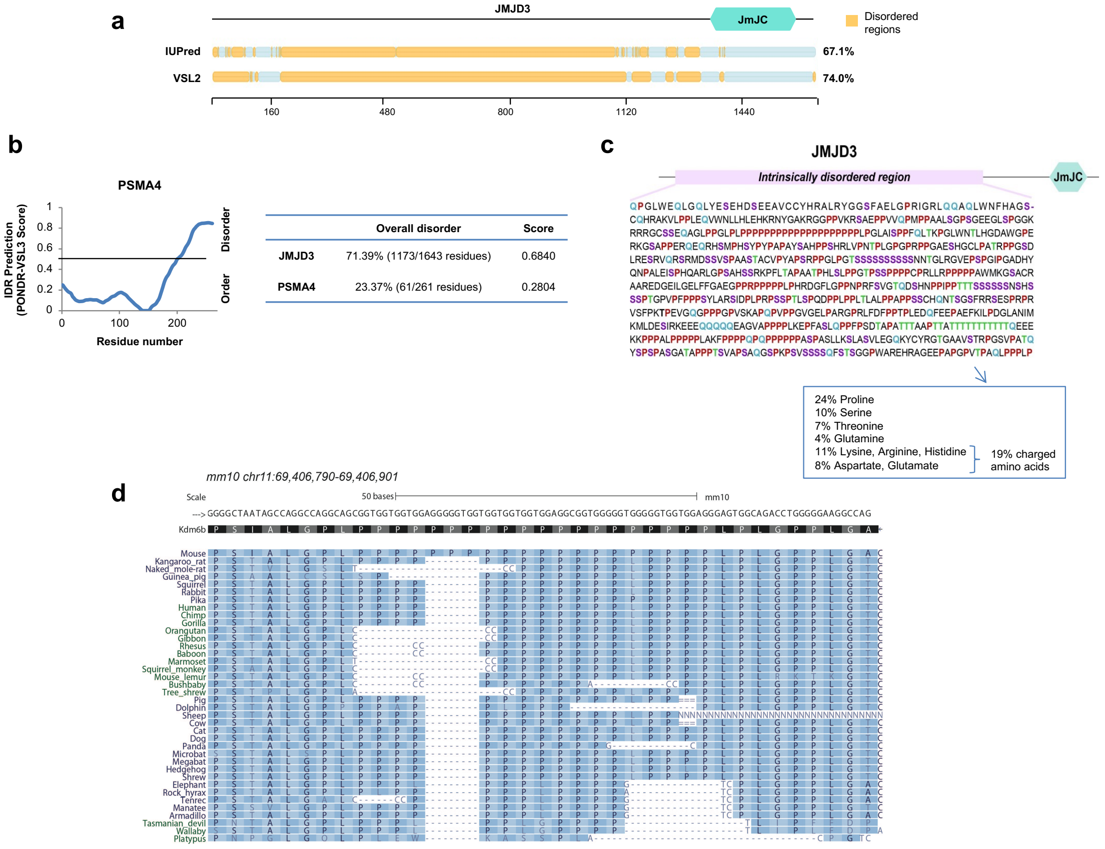

Supplementary Figure 6. **JMJD3 is a highly disordered protein. a** Disorder prediction of human JMJD3 using IUPred and VSL2 algorithms (see methods). The percentage of predicted disordered regions are indicated on the right. The disordered regions are marked in yellow (length of disordered segments > 30 amino acids). **b** Disorder prediction of PSMA4 using PONDR-VSL3 algorithm (see methods). At the bottom panel, the disorder score and the lengths of the predicted disordered regions are indicated (length of disordered segments > 50 amino acids). **c** Amino acid composition of JMJD3. The percentage of prolines (red), serines (purple), threonines (green), glutamines (light

blue), as well as basic and acidic amino acids are indicated at the bottom part of the panel. **d** UCSC tracks displaying the conservation of the proline-rich region of JMJD3 among vertebrate species using Multiz alignments[4].

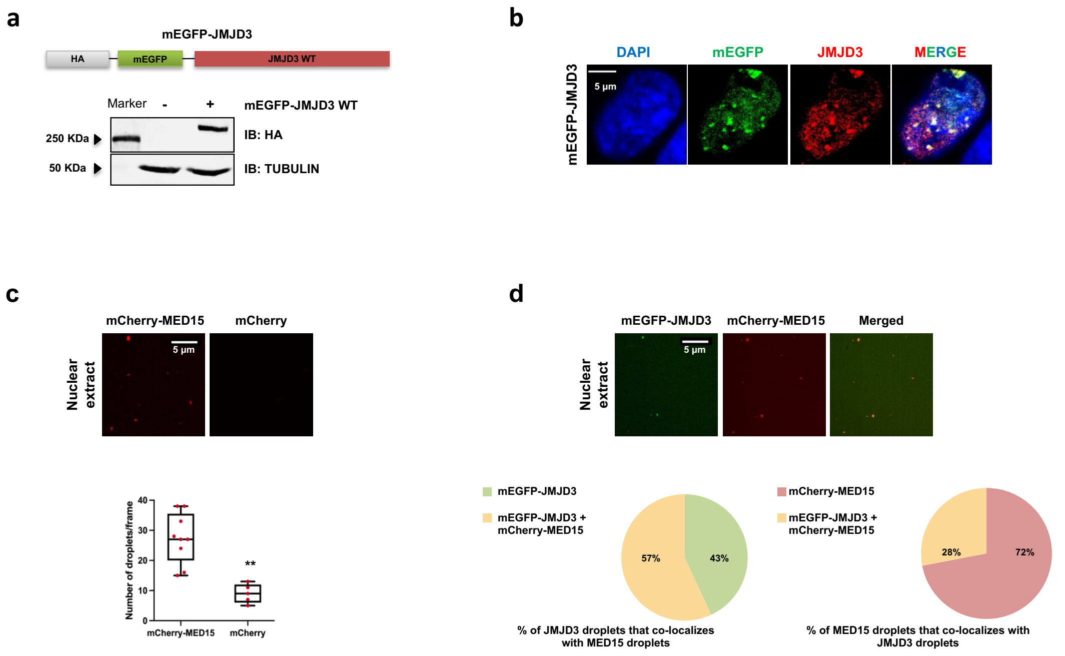

Supplementary Figure 7. **JMJD3 phase separates and its condensates colocalize with MED15**. **a** mEGFP–JMJD3 expression vector was transfected (0.05ug) in HEK293T. 24 h later, total protein extracts were prepared and the JMJD3 and TUBULIN levels were determined by immunoblot using HA-tag and TUBULIN antibodies respectively. Depicted result is representative of three biological replicates with similar results. Source data are provided as a Source Data file. **b** Confocal microscopy images of HEK293T cells transfected with 0.05ug mEGFP-JMJD3 and stained with JMJD3 antibody. DNA was visualized with DAPI staining. Merged image shows the overlap between the mEGFP signal and JMJD3 staining. The images are representatives of three biologically independent experiments. Scale bar, 5 μm. **c** mCherry-MED15 and mCherry proteins were used in droplet-formation assays in nuclear extracts at room temperature in the

presence of 150 mM NaCl. Quantifications of the number of droplets per frame are displayed. Data are the mean ± SEM. Boxes comprise values from Q1 to Q3 of the dataset; line corresponds to median value; whiskers show the data range (from min. to max. values within dataset). **$p<0.01$ (P values were calculated using one-tailed Student's t-test, p= 0.00653323). Droplets in 5 fields in each group from three biologically independent experiments were quantified. Scale bar, 5 μm. Source data are provided as a Source Data file. **d** mCherry-MED15 and mEGFP–JMJD3 were used in a droplet-formation assays in nuclear extracts as described above. Diagrams showing the percentage of JMJD3 droplets that colocalizes with MED15 and vice-versa are displayed at the bottom of the figure. Presented results are representative of three biological experiments. Source data are provided as a Source Data file.

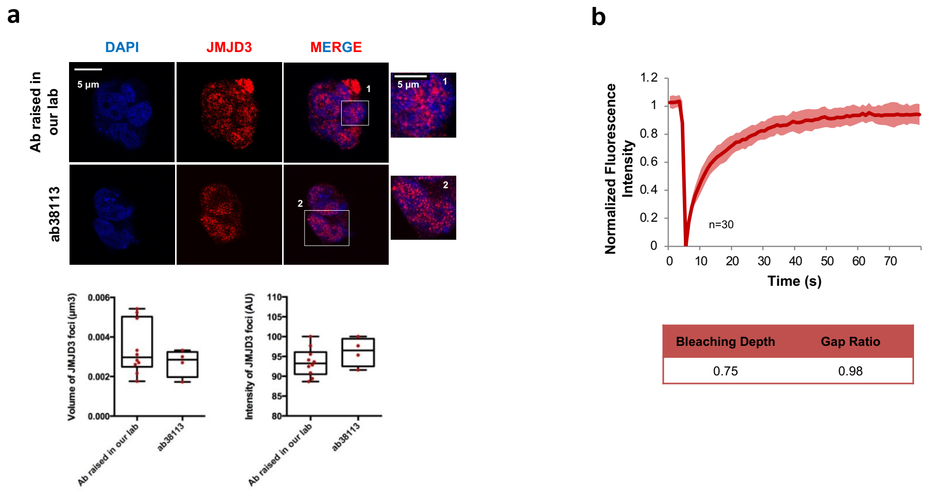

Supplementary Figure 8. **JMJD3 forms liquid-like condensates. a** HEK293T cells were fixed and endogenous JMJD3 was visualized by immunostaining assay using a

commercial antibody (ab38113) or an antibody risen in our laboratory[3]. Confocal microscopy images show the presence of JMJD3 puncta with endogenous protein levels. Smaller squares display zooms of the indicated regions. The images are representative of three biological independent experiments. Scale bar, 5 μm. Data are the mean ± SEM. Boxes comprise values from Q1 to Q3 of the dataset; line corresponds to median value; whiskers show the data range (from min. to max. values within dataset). Quantifications of the volume and intensity of JMJD3 puncta were performed for *n* =10 transfected cells. Source data are provided as a Source Data file. **b** Quantification of FRAP data for mEGFP-JMJD3, where bleaching events occur at t=4s with 82% loss of fluorescence in the bleaching region (bleaching depth) and 97% of total fluorescence remaining in the cell (gap ratio). Background-subtracted and normalized fluorescence intensities are plotted relative to a pre-bleach time point (t=0s). Data are plotted as mean normalized +/- SD (n=30 cells). Source data are provided as a Source Data file.

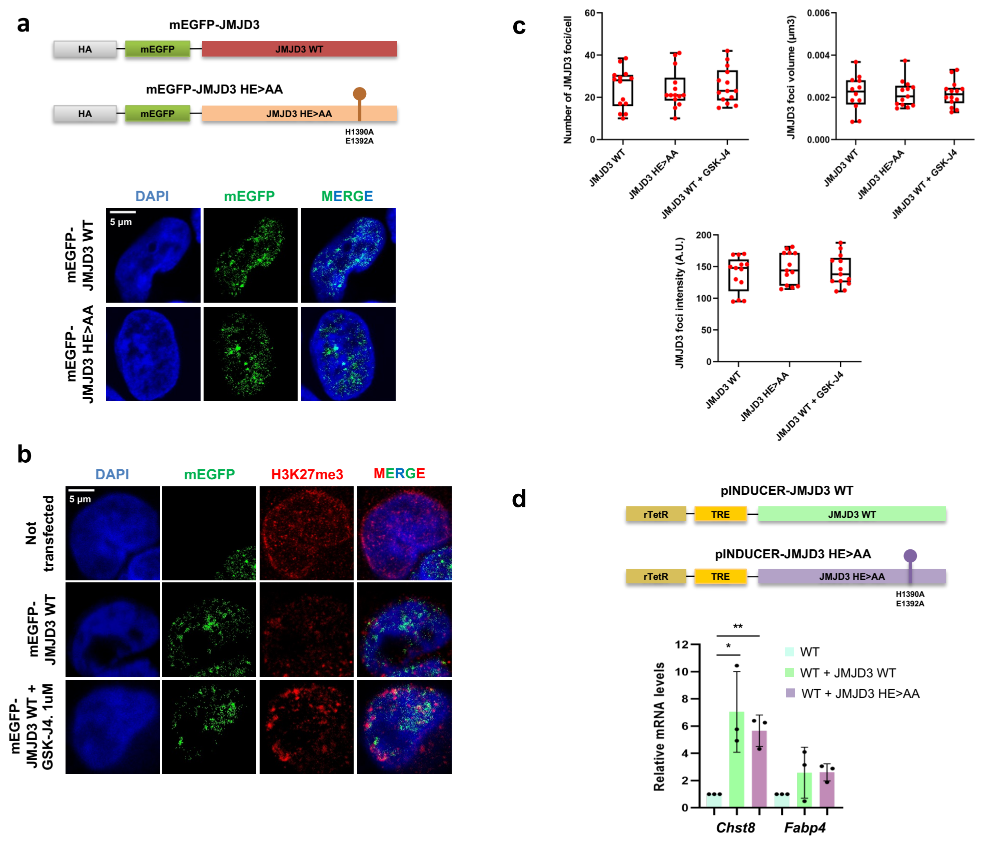

Supplementary Figure 9. **JMJD3 catalytic activity is not required for condensate formation. a** Confocal microscopy images of HEK293T cells transfected with 0.05ug mEGFP-JMJD3 WT or mEGFP-JMJD3 HE>AA (see top of the figure). The images are representatives of three biologically independent experiments. Quantifications of the number, volume and intensity of JMJD3 puncta are shown in (c). Scale bar, 5 μm. **b** Confocal microscopy images of HEK293T cells not transfected or transfected with 0.05ug mEGFP-JMJD3WT untreated or treated with 1uM GSK-J4 for 6 hours and immunostained with H3K27me3 antibody. DAPI was used to visualize the DNA. The images are representatives of three biologically independent experiments. Quantifications of the number, volume and intensity of JMJD3 puncta are shown in (c). Scale bar, 5 μm. **c** Quantifications of the number, volume and intensity of JMJD3 puncta obtained in (a) and (b). Data show the mean ± SEM. Boxes comprise values from Q1 to Q3 of the dataset; line corresponds to median value; whiskers show the data range (from min. to max. values within dataset). *n* =30 transfected cells in each group were quantified. Source data are provided as a Source Data file. **d** JMJD3 WT or JMJD3 HE>AA were expressed in HEK293T cells where the TGFβ pathway is active. Total RNA was prepared and the expression levels of *Chst8* mRNA were determined by qPCR. mRNA of *Fapb4* was used as a control. Transcription values were normalized to the housekeeping gene *Gapdh*, and figure shows values relative to WT line. Results are representatives of six technical replicate experiments. Data are the mean ± SEM. *p<0.05, **p<0.01 (P values were calculated using two-tailed Student's t-test, p= 0.024364077 and p= 0.002224677). Source data are provided as a Source Data file.

Supplementary Table 1: List of primers used in this study

	Region	Forward primer (FW)	Reverse primer (RV)
**cDNA**	*JMJD3* mRNA	CTCGTTCTGAGTCTGAGGT	CCCGCTCTACAAGGGTC
	Chst8 mRNA	CTTTCCCGAGGTTCAAGGAC	GGTCCGAGAAAGGTTTGGAG
	Pepd mRNA	CGAGGGCATTAGCAAGTTCA	AGCGCAAAACCTCTAGTTCC
	Fabp4 mRNA	TGAGTCCCCCACTTGCTTTA	CACCCTGTAAGGCTGGTGAT
	Fabp4 eRNA	TGAGTCCCCCACTTGCTTTA	CACCCTGTAAGGCTGGTGAT
	Chst8 Viewpoint (VP) eRNA	TAGGAGTGGACCAGTTAGC	CTTCTCTTCCCACCGTCAA
	Chst8 Enhancer 1 (E1) eRNA	GAGACAGTCGCCTTCCTGTC	TGGGATCTTGATGGCTACCT
	Chst8 Enhancer 2 (E2) eRNA	GGTTGTGGTCAGGGTG	ACTGGGCAAAGAATCTATGG
	Chst8 Enhancer 3 (E3) eRNA	GAGCAGTTCACCTAGATATT	TGTAACTGTAGGATACATGG
	Chst8 Enhancer 4 (E4) eRNA	TGACTCACTGGCTTGGTCAG	TGTCTGGCTGCAGGTCATAG
**ChIP**	*Chst8* promoter	ACTACAGTACGCGTGGAATT	GAGTCCCGGTTCTACACTTG
	Hoxd8 promoter	CAGTCTCTGGCAGTTCTTT	CCTGTCCTGTGCTTAACG
	Ctgf enhancer	TCACGCTGCTCCCTT	CTCTTGTCTTCCTCTGCTATG
	Chst8 Viewpoint (VP) enhancer	TAGGAGTGGACCAGTTAGC	CTTCTCTTCCCACCGTCAA
	Chst8 Enhancer 1 (E1)	GAGACAGTCGCCTTCCTGTC	TGGGATCTTGATGGCTACCT
	Chst8 Enhancer 2 (E2)	GGTTGTGGTCAGGGTG	ACTGGGCAAAGAATCTATGG
	Chst8 Enhancer 3 (E3)	GAGCAGTTCACCTAGATATT	TGTAACTGTAGGATACATGG
	Chst8 Enhancer 4 (E4)	TGACTCACTGGCTTGGTCAG	TGTCTGGCTGCAGGTCATAG
**Cloning for overexpression**	mEGFP	AGCTGAGCAAAGACCCCAACG	TGGACTGGGTGCTCAGGTAGT
**CRISPR-Cas9**	*Chst8* Enhancer left cut gRNAs	CACCGAACTGGGGTCACCCTCAAAG	AAACCTTTGAGGGTGACCCCAGTTC
	Chst8 Enhancer right cut gRNAs	CACCGACATTCAAAAGATTATGCGA	AAACTCGCATAATCTTTTGAATGTC
	Chst8 Enhancer test deletion (1+2)	CTGTCCTGTCCTCCTCCATC	GCAGCATCTCAGTCCAGGTC
	Chst8 Enhancer test deletion (1+3)	CTGTCCTGTCCTCCTCCATC	AAACGTTTCACCATGGAAGC

Supplementary Table 2: List of primers used in the 4C-seq assay

Region	Primer
FW *Chst8* shC 0h Replicate 1	AATGATACGGCGACCACCGAACACTCTTTCCCTACACGACGCTCTTCCGATCT**ACAGTGGGTTCCCCGTCATTCATG**
FW *Chst8* shC 3h Replicate 1	AATGATACGGCGACCACCGAACACTCTTTCCCTACACGACGCTCTTCCGATCT**GTGAAGGTTCCCCGTCATTCATG**
FW *Chst8* shJMJD3 3h Replicate 1	AATGATACGGCGACCACCGAACACTCTTTCCCTACACGACGCTCTTCCGATCT**CTTGGGTTCCCCGTCATTCATG**
FW *Chst8* shC 0h Replicate 2	AATGATACGGCGACCACCGAACACTCTTTCCCTACACGACGCTCTTCCGATCT**GCCGGTTCCCCGTCATTCATG**

FW *Chst8* shC 3h Replicate 2	AATGATACGGCGACCACCGAACACTCTTTCCCTACACGACGCTCTTCCGATCT**CGTACTGGTTCCCCGTCATTCATG**
FW *Chst8* shJMJD3 3h Replicate 2	AATGATACGGCGACCACCGAACACTCTTTCCCTACACGACGCTCTTCCGATCT**TAAGGGGTTCCCCGTCATTCATG**
FW *Chst8* shJMJD3 + JMJD3 3h Replicate 1	AATGATACGGCGACCACCGAACACTCTTTCCCTACACGACGCTCTTCCGATCT**AGGCGGTTCCCCGTCATTCATG**
FW *Chst8* shJMJD3 + JMJD3 3h Replicate 2	AATGATACGGCGACCACCGAACACTCTTTCCCTACACGACGCTCTTCCGATCT**TCGAACGGTTCCCCGTCATTCATG**
RV *Chst8*	CAAGCAGAAGACGGCATACGA**CCAGCTGGTTAACACAGGA**
FW *Ldlrad4* shC 0h Replicate 1	AATGATACGGCGACCACCGAACACTCTTTCCCTACACGACGCTCTTCCGATCT**ACAGTGAGAACACCAGAGTAGGCATG**
FW *Ldlrad4* shC 3h Replicate 1	AATGATACGGCGACCACCGAACACTCTTTCCCTACACGACGCTCTTCCGATCT**GTGAAAGAACACCAGAGTAGGCATG**
FW *Ldlrad4* shJMJD3 3h Replicate 1	AATGATACGGCGACCACCGAACACTCTTTCCCTACACGACGCTCTTCCGATCT**CTTGAGAACACCAGAGTAGGCATG**
FW *Ldlrad4* shC 0h Replicate 2	AATGATACGGCGACCACCGAACACTCTTTCCCTACACGACGCTCTTCCGATCT**GCCAGAACACCAGAGTAGGCATG**
FW *Ldlrad4* shC 3h Replicate 2	AATGATACGGCGACCACCGAACACTCTTTCCCTACACGACGCTCTTCCGATCT**CGTACTAGAACACCAGAGTAGGCATG**
FW *Ldlrad4* shJMJD3 3h Replicate 2	AATGATACGGCGACCACCGAACACTCTTTCCCTACACGACGCTCTTCCGATCT**TAAGGAGAACACCAGAGTAGGCATG**
RV *Ldlrad4*	CAAGCAGAAGACGGCATACGA**CTTGCTCAAGTCGCTCGC**
FW *Aopep* shC 0h Replicate 1	AATGATACGGCGACCACCGAACACTCTTTCCCTACACGACGCTCTTCCGATCT**ACAGTGTCACAGTATGGGAATCATG**
FW *Aopep* shC 3h Replicate 1	AATGATACGGCGACCACCGAACACTCTTTCCCTACACGACGCTCTTCCGATCT**GTGAATCACAGTATGGGAATCATG**
FW *Aopep* shJMJD3 3h Replicate 1	AATGATACGGCGACCACCGAACACTCTTTCCCTACACGACGCTCTTCCGATCT**CTTGTCACAGTATGGGAATCATG**
FW *Aopep* shC 0h Replicate 2	AATGATACGGCGACCACCGAACACTCTTTCCCTACACGACGCTCTTCCGATCT**GCCTCACAGTATGGGAATCATG**
FW *Aopep* shC 3h Replicate 2	AATGATACGGCGACCACCGAACACTCTTTCCCTACACGACGCTCTTCCGATCT**CGTACTTCACAGTATGGGAATCATG**
FW *Aopep* shJMJD3 3h Replicate 2	AATGATACGGCGACCACCGAACACTCTTTCCCTACACGACGCTCTTCCGATCT**TAAGGTCACAGTATGGGAATCATG**
RV *Aopep*	CAAGCAGAAGACGGCATACGA**CATCACTACGGGAAAGTCA**

Supplementary Table 3: List of genome-wide data accessions used in this paper

Data	Accession number
SMAD3 0,5h TGFβ ChIP-seq	GSM898371
JMJD3 3h TGFβ ChIP-seq	GSM937827
H3K27ac ChIP-seq	GSE66961
H3K4me1 ChIP-seq	GSE66961
H3K4me3 ChIP-seq	GSE66961
SMC1 ChIP-seq	GSM883646
H3K27me3 ChIP-seq	GSE38269

SUPPLEMENTARY REFERENCES

1. Estarás, C. *et al*. Genome-wide analysis reveals that Smad3 and JMJD3 HDM co-activate the neural developmental program. *Development* **139,** 2681–2691 (2012).

2. Kent, W. J. *et al*. The Human Genome Browser at UCSC. *Genome Res*. **12,** 996–1006 (2002).

3. Fueyo, R. *et al*. Lineage specific transcription factors and epigenetic regulators mediate TGFβ-dependent enhancer activation. *Nucleic Acids Res*. **46,** 3351–3365 (2018).

4. Blanchette, M. *et al*. Aligning Multiple Genomic Sequences With the Threaded Blockset Aligner. *Genome Res*. **14,** 708–715 (2004).
