# Supplementary material for: JMJD3 intrinsically disordered region links the 3D-genome structure to TGFβ-dependent transcription activation
Source: Nat Commun. 2022 Jun 7;13:3263. doi: 10.1038/s41467-022-30614-y (PMC9174158; doi:10.1038/s41467-022-30614-y)
Supplement: Supplementary file 2 — Description of additional Supplementary File [file 41467_2022_30614_MOESM2_ESM.pdf]

Descriptions of additional supplementary files

Supplementary Movie S1. FRAPexperiment in which the quick recovery of mEGFP-JMJD3 puncta after photobleaching is observed.

Supplementary Movie S2. FRAP experiment in which the aggregation of mEGFP-JMJD3 is appreciated by means of a reduced mobility after photobleaching.

Supplementary Data file 1. Table depicting the parameters that assess the quality of the Chst8 4C-seq experiments for each sample as previously described. Briefly, the % of reads containing VP sequence should be above 90, the % of fragments that map in cis above 50, and the % of reads that map in unique sites within 1Mb around VP above 60.

Supplementary Data file 2 and 3. Tables containing the quality measurements for each sample of the Ldlrad4 4C-seq (2) and Aopep 4C-seq (3) experiments, according to the parameters previously described. Briefly, the % of reads containing VP sequence should be above 90, the % of fragments that map in cis above 50, and the % of reads that map in unique sites within 1Mb around VP above 60.
