# Supplementary material for: JMJD3 intrinsically disordered region links the 3D-genome structure to TGFβ-dependent transcription activation
Source: Nat Commun. 2022 Jun 7;13:3263. doi: 10.1038/s41467-022-30614-y (PMC9174158; doi:10.1038/s41467-022-30614-y)
Supplement: Supplementary file 8 — Reporting Summary [file 41467_2022_30614_MOESM8_ESM.pdf]

nature portfolio

Corresponding author(s): M Martinez-Balbas

Last updated by author(s): Apr 20, 2022

Reporting Summary

Nature Portfolio wishes to improve the reproducibility of the work that we publish. This form provides structure for consistency and transparency in reporting. For further information on Nature Portfolio policies, see our Editorial Policies and the Editorial Policy Checklist.

Statistics

For all statistical analyses, confirm that the following items are present in the figure legend, table legend, main text, or Methods section.

n/a	Confirmed	
☐	☒	The exact sample size (n) for each experimental group/condition, given as a discrete number and unit of measurement
☐	☒	A statement on whether measurements were taken from distinct samples or whether the same sample was measured repeatedly
☐	☒	The statistical test(s) used AND whether they are one- or two-sided<br>*Only common tests should be described solely by name; describe more complex techniques in the Methods section.*
☒	☐	A description of all covariates tested
☒	☐	A description of any assumptions or corrections, such as tests of normality and adjustment for multiple comparisons
☐	☒	A full description of the statistical parameters including central tendency (e.g. means) or other basic estimates (e.g. regression coefficient) AND variation (e.g. standard deviation) or associated estimates of uncertainty (e.g. confidence intervals)
☐	☒	For null hypothesis testing, the test statistic (e.g. F, t, r) with confidence intervals, effect sizes, degrees of freedom and P value noted<br>*Give P values as exact values whenever suitable.*
☒	☐	For Bayesian analysis, information on the choice of priors and Markov chain Monte Carlo settings
☒	☐	For hierarchical and complex designs, identification of the appropriate level for tests and full reporting of outcomes
☒	☐	Estimates of effect sizes (e.g. Cohen's d, Pearson's r), indicating how they were calculated

Our web collection on statistics for biologists contains articles on many of the points above.

Software and code

Policy information about availability of computer code

Data collection	We used Zen software (Zen black version 8,1,0,484) from Zeiss to collect confocal images in this study.

Data analysis	4C-seq and ChIP-seq data:<br>4C-seq sequences were processed using the 4C-seq pipeline named pipe4C (Krijger et al., 2020) using default parameters except for the trimLength that was set up to 36 bp, and the genome version for mapping that was Mus musculus mm10. Further statistical analysis was performed with R3Cseq (Thongjuea S et al., 2013), a Bioconductor package that allows the identification of interacting genomic regions and the comparison between multiple replicates and experimental conditions.<br>Both 4C and ChIP-seq captions were obtained from UCSC genome browser (Kent et al., 2002)<br><br>Protein sequence features and predictions:<br>Protein disorder estimations were generated using three prediction algorithms, PONDRVL3 (Peng et al., 2005), IUPred (Dosztányi et al., 2005) and PONDR-VSL2 (Peng et al.,2006)<br>Phase separation propensity was predicted with PSPredictor (Sun et al., 2020) and catGRANULE (Bolognesi et al., 2016)<br>Low-complexity domains presence was assessed using the SEG algorithm together with the MobiDB database (Piovesan et al., 2018).<br>Amino acid composition was analysed using the web application Prot Pi Protein Tool (https://www.protpi.ch/Calculator/ProteinTool)<br>Hydrophobicity was calculated with the ExPASy website (Gasteiger et al., 2003)<br><br>FRAP data:<br>We have used a custom macro that has been deposited in GitHub (https://github.com/MolecularImagingPlatformIBMB)<br><br>Image analysis (immunofluorescence and live-cell imaging):<br>We have used Fiji software (version 2015 December 22, Java 6) (Schindelin et al., 2009)

For manuscripts utilizing custom algorithms or software that are central to the research but not yet described in published literature, software must be made available to editors and reviewers. We strongly encourage code deposition in a community repository (e.g. GitHub). See the Nature Portfolio guidelines for submitting code & software for further information.

Data

Policy information about availability of data

All manuscripts must include a data availability statement. This statement should provide the following information, where applicable:

- Accession codes, unique identifiers, or web links for publicly available datasets
- A description of any restrictions on data availability
- For clinical datasets or third party data, please ensure that the statement adheres to our policy

All 4C-seq data that N the findings of this study are available at GEO under the accession code GSE197013 (https://www.ncbi.nlm.nih.gov/geo/query/acc.cgi?acc=GSE197013).
The ChIP-seq data that support the findings of this study are available in GEO with the identifier(s) GSM898371 (https://www.ncbi.nlm.nih.gov/geo/query/acc.cgi?acc=GSM898371), GSM937827 (https://www.ncbi.nlm.nih.gov/geo/query/acc.cgi?acc=GSM937827), GSE66961 (https://www.ncbi.nlm.nih.gov/geo/query/acc.cgi?acc=GSE66961), GSE66961 (https://www.ncbi.nlm.nih.gov/geo/query/acc.cgi?acc=GSE66961), GSE66961 (https://www.ncbi.nlm.nih.gov/geo/query/acc.cgi?acc=GSE66961), GSM883646 (https://www.ncbi.nlm.nih.gov/geo/query/acc.cgi?acc=GSM883646), GSE38269 (https://www.ncbi.nlm.nih.gov/geo/query/acc.cgi?acc=GSE38269)
All relevant data supporting the key finding of this work are available in Supplementary Information and within the article. Source data are provided with this paper.

Field-specific reporting

Please select the one below that is the best fit for your research. If you are not sure, read the appropriate sections before making your selection.

☒ Life sciences ☐ Behavioural & social sciences ☐ Ecological, evolutionary & environmental sciences

For a reference copy of the document with all sections, see nature.com/documents/nr-reporting-summary-flat.pdf

Life sciences study design

All studies must disclose on these points even when the disclosure is negative.

Sample size	The sample size was estimated from the preliminary experiments or from relevant previously published studies (Zamudio, A. V. et al. Mol. Cell (2019); Nair, S.J.et al. Nat Struct Mol Biol (2019); Cai, D. et al. Nat Cell Biol (2019)). No statistical method was applied to predetermine sample size. The sample size is sufficient since we usually detect large difference between two experimental conditions, with p-value lower than 0.01. The RT-qPCR, ChIP-qPCR and imaging assays were conducted with three independent experiments or samples. 4c-seq, PCR and Western Blot assays were conducted with two independent samples, and one representative plot is shown in figures. Most P values were obtained using a two-tailed Student's t-test. The number of nuclei examined and statistical test used for microscopy data analysis are provided with each data in the figures.
Data exclusions	No data were excluded from the analyses
Replication	Most of the experiments were performed at least in triplicate, and some in duplicate. When all experiments were successful we concluded that experiment was reproducible and included it in the study
Randomization	Not relevant to the study, as in our study the main experimental groups were:

Randomization	- wild type or control versus knock down or knock out samples<br>- wild type or control versus mutant samples<br>- non-treated versus treated samples (TGβ, 1,6-Hexanediol, doxycycline, GSK-J4 treatments)
Blinding	In our study blinding was not relevant as we allocated samples into pre-determined experimental groups with the intention of studying the differences between them.

Reporting for specific materials, systems and methods

We require information from authors about some types of materials, experimental systems and methods used in many studies. Here, indicate whether each material, system or method listed is relevant to your study. If you are not sure if a list item applies to your research, read the appropriate section before selecting a response.

Materials & experimental systems

n/a	Involved in the study	
☐	☒	Antibodies
☐	☒	Eukaryotic cell lines
☒	☐	Palaeontology and archaeology
☒	☐	Animals and other organisms
☒	☐	Human research participants
☒	☐	Clinical data
☒	☐	Dual use research of concern

Methods

n/a	Involved in the study	
☒	☐	ChIP-seq
☒	☐	Flow cytometry
☒	☐	MRI-based neuroimaging

Antibodies

Antibodies used	- JMJD3: raised in the laboratory, using amino acids 798–1095<br>- JMJD3: Abcam, ab38113, Lot: GR3228142-1<br>- DAPI: ThermoFisher, D1306, Lot: 1023584<br>- β-TUBULIN: Millipore, MAB3408, clone KMX1, Lot: 2918041<br>- HA tag: Abcam, ab20084, Lot: GR2051-3<br>- H3K9me3: Abcam, ab8898, Lot: GR131093-3<br>- H3K27me3: Millipore, 07-449, Lot: 3574311<br>- Alexa anti-rabbit 488: Invitrogen, A32731, Lot: 1575605<br>- Alexa anti-rabbit 555: Invitrogen, A32732, Lot: 1454443
Validation	- JMJD3: validated for Western blot in Estarás et al., 2012; Estarás et al., 2013; Fueyo et al., 2018. Validated for immunofluorescence in the present study (used again endogenous and overexpressed protein).<br>- JMJD3: validated for immunofluorescence in Abcam website (https://www.abcam.com/kdm6b-jmjd3-antibody-ab38113.html).<br>- DAPI: validated for immunofluorescence in many citations that can be found in manufacturer’s website (https://www.thermofisher.com/order/catalog/product/D1306#/D1306). Also validated in many papers done in our lab, such as Fueyo et al., 2018.<br>- β-TUBULIN: validated for western blot in manufacturer’s website (https://www.merckmillipore.com/ES/es/product/Anti-Tubulin-Antibody-beta-clone-KMX-1,MM_NF-MAB3408?ReferrerURL=https%3A%2F%2Fwww.google.com%2F&bd=1).<br>- HA tag: validated for western blot in manufacturer’s website (https://www.abcam.com/ha-tag-antibody-ab20084.html).<br>- H3K9me3: validated for immunofluorescence in manufacturer’s website (https://www.abcam.com/histone-h3-tri-methyl-k9-antibody-chip-grade-ab8898.html).<br>- H3K27me3: validated for IP in manufacturer’s website (https://https://www.merckmillipore.com/ES/es/product/Anti-trimethyl-Histone-H3-Lys27-Antibody,MM_NF-07-449).<br>- Alexa anti-rabbit 488: validated for immunofluorescence in manufacturer's website (https://www.thermofisher.com/antibody/product/Goat-anti-Rabbit-IgG-H-L-Highly-Cross-Adsorbed-Secondary-Antibody-Polyclonal/A32731)<br>- Alexa anti-rabbit 555: validated for immunofluorescence in manufacturer's website (https://www.thermofisher.com/antibody/product/Goat-anti-Rabbit-IgG-H-L-Highly-Cross-Adsorbed-Secondary-Antibody-Polyclonal/A32732)

Eukaryotic cell lines

Policy information about cell lines

Cell line source(s)	Neural Stem Cells (NSCs) were extracted from cerebral cortices of C57BL/6J mouse embryos from E12.5 (Estarás, C. et al. Development (2012)), and since then are cultured as an estable cell line.<br><br>HEK293T cells used were from ATCC (ATTC Cat# CRL-3216, RRID: CVCL_0063)
Authentication	None of the cells used were authenticated
Mycoplasma contamination	Cell lines were not tested for mycoplasma contamination

Commonly misidentified lines<br>(See ICLAC register)	No commonly misidentified cell lines were used in the study
